# Epoxidation of Alkenes by Peracids: From Textbook Mechanisms to a Quantum Mechanically Derived Curly‐Arrow Depiction

**DOI:** 10.1002/open.201900099

**Published:** 2019-07-12

**Authors:** Johannes E. M. N. Klein, Gerald Knizia, Henry S. Rzepa

**Affiliations:** ^1^ Molecular Inorganic Chemistry, Stratingh Institute for Chemistry, Faculty of Science and Engineering University of Groningen Nijenborgh 4 9747 AG Groningen The Netherlands; ^2^ Department of Chemistry Pennsylvania State University 401A Chemistry Bldg; University Park PA 16802 USA; ^3^ Department of Chemistry Imperial College London, MSRH 80 Wood Lane London W12 0BZ UK

## Abstract

Using the intrinsic bond orbital (IBO) analysis based on accurate quantum mechanical calculations of the reaction path for the epoxidation of propene using peroxyacetic acid, we find that the four commonly used curly arrows for representing this reaction mechanism are insufficient and that seven curly arrows are required as a result of changes to σ and π bonding interactions, which are usually neglected in all textbook curly arrow representations. The IBO method provides a convenient quantitative method for deriving curly arrows in a rational manner rather than the normal *ad hoc* representations used ubiquitously in teaching organic chemistry.

## 1 Introduction

Curly arrow depictions^1^, ^2^, ^3^ of how bonds break and form during chemical transformations have been ubiquitous in both textbooks and research articles for seventy years or more. Whilst Robinson is generally credited with introducing the formalism, the initial example^1^ was restricted to illustrating resonance in hexatriene and it is the better‐known second attempt in 1924^2^ to explain a reaction outcome that more closely resembles the modern mechanistic usage. Whilst most practitioners of curly arrows tend to assume they represent just a convenient formalism,^4^ efforts continue to extract more formal curly arrow descriptions from quantum chemistry calculations.^5^

**Scheme 1:**
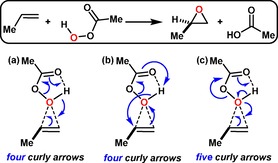
(a) Textbook curly arrow mechanism^7^ for alkene epoxidation with a style depicting the terminus bearing the arrow head as forming a covalent bond to the bond mid‐point and (b) alternative style^8^ depicting the termination of (some) arrow heads directly at an atom. Mechanistic alternative (c) uses five curly arrows.^9^

Although used intermittently in research articles during the 1930s and 1940s, the adoption of curly arrows to depict the mechanism of reactions accelerated in the 1950s, especially so when well‐known textbooks started to adopt them.^6^ Various styles evolved based on rules established by practice and attempted clarity rather than from a sound theoretical basis. A deceptively simple example of curly arrow depiction is the epoxidation reaction of alkenes by peracids. One common textbook description of this reaction is shown in Scheme 1(a),^7^ with four curly arrows indicating that four electron pairs will undergo significant changes during the course of what is implied as a concerted synchronous mechanism. These changes include (i) the nucleophilic attack of the C−C π‐bond onto the assumed electrophilic oxygen of the peracid unit, (ii) transformation of the O−H bond of the peracid unit into a new C−O bond of the epoxide product, (iii) breaking of the O−O bond in which the electron pair is transformed into the C=O bond of the carboxylic acid by product and (iv) transformation of the π‐bond of the C=O bond in the starting peracid into an O−H bond. For this particular mechanism at times the end‐point of the curly arrow indicating the formation of a given bond can differ such that the termination of (some) arrow heads ends directly at an atom (Scheme 1(b)).^4^, ^8^ An alternative depiction (Scheme 1(c))^9^ involves instead five curly arrows via oxygen lone pair participation, thus recognising the differing nucleophilic character of an oxygen lone pair versus a O−H covalent bond. Such variation illustrates the need for a more formal procedure for deciding the attributes of the arrows, which would include establishing the total curly arrow count, their direction and their choreography or timing.

It becomes quickly apparent that these curly arrow descriptions require the interconversion of σ and π bonds (as indeed does Robinson's^2^ example). Intrinsic bond orbitals (IBOs)^10^ are a form of localised orbital that have the property of evolving in a well‐behaved and continuous manner across the intrinsic reaction coordinate (IRC)^11^ of a reaction, unlike other localised orbital functions. We have previously demonstrated for the Claisen rearrangement^12^ that transformations between σ and π bonds can be easily identified when following reaction paths using IBOs. Here we focus on applying IBOs to the epoxidation reaction shown in Scheme 1, including the evolution of σ and π bonding and showing how the results can be mapped to the use of curly arrows. We will demonstrate that a full description of the changes to bonding for the epoxidation reaction of alkenes using peracids is possible using IBOs. To augment our studies we also highlight the importance of the changes to the dipole moment during the course of the IRC for these reactions as an indicator of “hidden” ionic intermediates during the course of the reaction.^13^

## 2 Results and Discussion

**Scheme 2:**
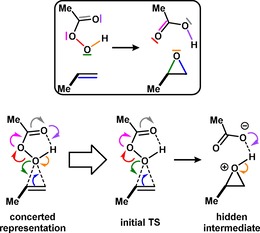
Curly arrows for the epoxidation of propene by peracetic acid as derived from the IBO analysis.

**Figure 1:**
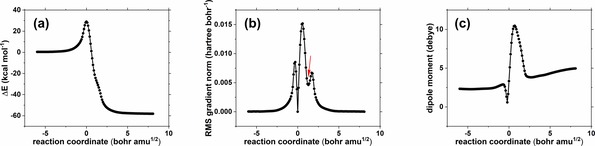
Intrinsic reaction coordinate for reaction of propene with peracetic acid at the M06‐2X/Def2‐TZVPPD/CPCM(CH_2_Cl_2_) level of theory showing (a) the energy profile, (b) the gradient norm profile illustrating the “hidden intermediate” at IRC=1.5 (see red arrow) and (c) the dipole moment response.

As a representative model system, we selected prop‐1‐ene and peracetic acid, as shown in Scheme 1, as a typical closed shell reaction involving only the transformation of electron pairs and taught in most courses of advanced organic chemistry (for the computational methods used see the Computational Details). Just as expected, we identified a single transition state which led to the formation of the desired epoxide product with a corresponding IRC followed from the transition state for this reaction that reveals both a complex formed at the end of the reaction with an intermolecular OH hydrogen bond and a “hidden intermediate” or HI^13^ (Scheme 2 and Figure 1(a) and 1(b)) manifesting only after the transition state is passed and corresponding to an unformed ionic species. This can be seen particularly well in the root mean square values of the gradient norm (Figure 1(b)), which temporarily decrease along the IRC and then increase again (see red arrow). The formation of a HI is followed by a proton transfer to complete the reaction, which reveals that the reaction coordinate is significantly asynchronous in character. This character is confirmed by the experimental kinetic isotope effects^14^ which show an inverse effect for ^2^H on the alkene indicating a change from sp^2^ to sp^3^ hybridisation at carbon due to C−O bond formation. A primary deuterium isotope effect would be expected from a transition state involving proton transfer.^9^ The dipole moment response along the IRC (Figure 1(c)) shows considerable, but temporary, charge reorganisation occurring immediately after the transition state is passed as the hidden ionic intermediate develops and then reforms a non‐ionic product resulting from proton transfer.

**Figure 2:**
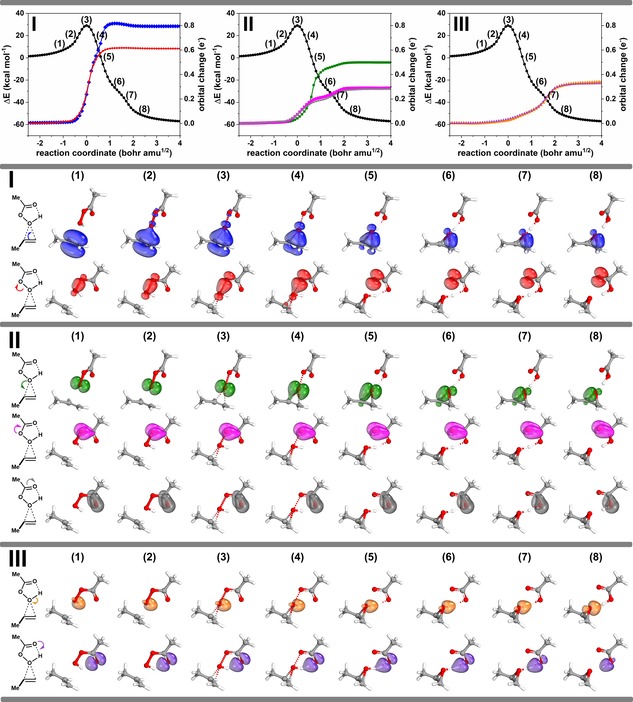
IBO changes connected to curly arrows, indicating the three different groupings **I–III** of electron pairs. See main text for definition of the quantity orbital change used in the top row.

The analysis of the IBOs in a straightforward fashion allows us to inspect how bonds are broken and made in this reaction. For this purpose, we simply take the Kohn‐Sham wave functions along every step of the IRC and transform the occupied molecular orbitals into IBOs. These localised orbitals now provide a direct link between the computational results and Lewis structure depictions. To monitor the changes of the IBOs and by extension bonds and lone pairs, we match IBOs between frames with the maximum overlap. In order to probe if an IBO and therefore a bond/lone pair is involved in a bond making or breaking event, we compute and plot the quantity orbital change (see Figure 2, top row), which compactly summarises the degree to which an orbital's electron charge distribution across atoms changes as the reaction progresses along the IRC. Orbital change is measured in units of electron charge (e^−^), and for orbital i at arc length s of the IRC concretely defined as

$$\Delta_{i}\left(s\right):=\sqrt{\sum_{A=1}^{atoms}{\left(n_{A,i}\left(s\right)-n_{A,i}\left(0\right)\right)}^{2}}$$

where A sums over the atoms and $n_{A,i}\left(s\right)$ is the number of electrons on atom A of orbital i at IRC arc length s –i. e., $\Delta_{i}\left(s\right)$ is the root square deviation of the atomic electron charge distribution at point s of the IRC versus the initial distribution (s=0). This criterion simplifies the identification of IBOs that are to be inspected in a given transformation. This procedure is automated in the IboView code^15^ and renders the overall process straightforward.

After we carried this procedure out, our analysis reveals that there are *seven* IBOs which are involved in changes, unlike the standard textbook curly arrow formalisms. So where does this discrepancy originate?

The answer to this question is rather simple and is captured by the single word: orientation. We have grouped the seven IBOs, and by extension the seven curly arrows, into three groups (Scheme 2), using colour coding:

- The alkene π bond *(*blue*)* and the O−O σ bond of the peracid (red),
- The π lone pair on the O−H oxygen of the peracid (green), the changes from a π lone pair to a true C=O π bond and the reverse (magenta and grey),
- Breaking and making of the O−H bond in the starting material and product (orange and purple). In Scheme 2 and Figure 2 we also use the same colour codes to highlight between these three groups of IBO changes.

Group **I** involves the transformation of the alkene π bond to one of the C−O bonds in the epoxide unit (blue) and the cleavage of the O−O σ bond of the peracid (red). Inspecting the IBOs it becomes clear that the alkene, much as expected, acts as a nucleophile and that the electron pair of this bond is rearranged into becoming a covalent C−O σ bond in the epoxide. Also, in this group is the heterolytic cleavage of the O−O σ bond resulting in the formation of a lone pair localised on what is to become the carbonyl C=O oxygen atom. However, unlike in the textbook mechanism, this does not become part of the delocalised π bonding regime. Figure 2 makes this clear as it shows that the orientation of the newly formed lone pair is oriented perpendicular to what will become the C=O π bond (vide infra). These two changes occur in a concerted fashion.

Subsequent changes to the bonding occur in group **II**. The most prominent is the formation of the second C−O σ bond of the epoxide, which is formed from a lone pair on the O−H oxygen atom (green). This already departs from the four‐arrow textbook mechanism, where the electron pair of the O−H σ bond is conventionally used for the C−O bond formation in the epoxide, matching instead the five‐arrow variation. This bond change is delayed compared the bond changes in group **I**, which may be attributed to the formation of the “hidden intermediate” which is stabilised by contribution of carbocation resonance character at the Me‐substituted carbon atom. In addition, this bond formation coincides with the change from an O‐centred π lone pair to the C=O π bond (magenta) and the opposite process (grey).

The final changes in group **III** conclude the epoxide formation, where the two other changes to bonds/lone pairs are associated with an intramolecular proton transfer. As the O−O bond breaks and the epoxide forms, the O−H bond (orange) morphs into a lone pair on the oxygen atom of the epoxide and the proton is picked up by a lone pair on the oxygen atom (purple) originally associated with the carbonyl oxygen atom of the peracid. Again, the orientation of the lone pairs is such that they do not allow for participation in delocalisation in the π system of the carboxylate unit, as implied by the textbook mechanism.

Overall, these changes identified by the IBO analysis of the electron flow only partially agree with conventional textbook mechanisms. It is crucial to appreciate that the major differences are a result of the orientation of bonds and Ione pairs featuring π symmetry and their changes. One could argue that this is a subtlety of minor relevance. So, *does* orientation matter*?* The question is of course rhetorical and the answer is *yes*. We may use the phenyl anion to make this point clear. In the anion, the carbon‐centred lone pair of the carbanion is localised and may not be drawn as part of the mesomeric structures used to represent the aromatic ring. The argument for this is simply that the orientation of this lone pair is perpendicular to the 6‐electron π system of the aromatic ring. In exactly the same way such a difference is found in the reaction between alkenes and peracids. The textbook representations do not take this subtle difference into account and therefore fail to provide even a qualitative description of the underlying changes to the electronic structure. One could argue that curly arrows are merely a formalism^4^, ^16^ and that they were not intended to capture the intricate changes that the electronic structure undergoes during a reaction. However, we here argue the exact opposite, demonstrating that the essence of curly arrows can indeed be recovered from quantum chemistry calculations using IBOs. Notably, in the same spirit others have also explored the connection between quantum chemistry and the curly arrow formalism, with a number of reports demonstrating this connection.^5^

If we accept this connection, there are some important conclusions that can be made. In particular, the orientation of IBOs, or more generally the orientation of bonding interactions, is fundamentally important, but is often not captured by curly arrows. If, however, we pay close attention we can indeed devise curly arrow mechanisms that are meaningful representations of electron flow in chemical reactions. An informally taught rule that should therefore be viewed with great caution is that in curly arrow representations, the mechanism represented by the least number of curly arrows may be the most appropriate. This latter statement inadvertently renders curly arrows a formalism, which they do not have to be.

Until this point, we have inspected the changes of the IBOs and their connection to curly arrows with little focus on how these changes are choreographed. In Figure 2 (top) we show plots of the orbital change quantity introduced above, which characterises how the initial partial charge distribution of a given IBO changes along the reaction path. When plotted along the reaction coordinate, this criterion allows us to judge how concerted these changes are. In the present case we can see in group **I** that the two C−O bonds in the epoxide are not formed in a synchronous fashion. Instead, the initial C−O bond is formed upon nucleophilic attack of the alkene onto the electrophilic oxygen, which occurs with an S_N_2‐like concomitant O−O bond scission. The presence of the Me substituent allows for the development of some temporary carbocation character, leading to a delay in the formation of the second C−O bond of the epoxide. As the O−O bond scission occurs in a concerted fashion, plot **II** indicates that the proton transfer occurs only after the epoxide is fully formed. Although the reaction proceeds with a single transition state, we can consider structure (**6**) in Figure 2 as a hidden intermediate (*c.f*. Scheme 2). Thus, epoxide formation occurs in a concerted asynchronous fashion leading to a hidden intermediate (**6**), which results in the formation of the product upon intramolecular proton transfer.

**Figure 3:**
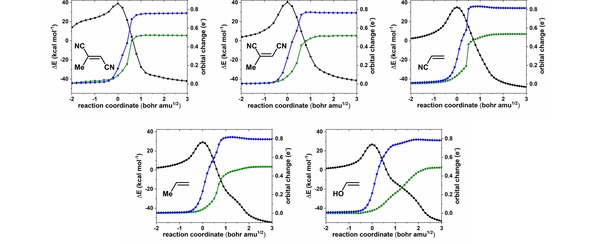
Changes in energy and IBO orbital change along the IRC for R=Me, OH, mono and cis and trans dicyano. See main text for definition of the quantity orbital change used in the top row.

**Figure 4:**
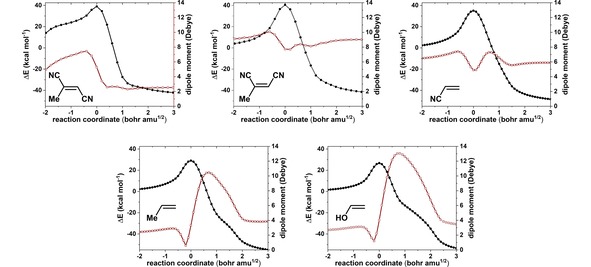
Changes in energy and dipole moments along the IRC for R=Me, OH, mono and cis and trans dicyano.

Through the use of IBOs we have not only shown that an accurate description of this reaction requires an extension of the textbook mechanism to seven curly arrows, but also elaborated the sequence of bond‐forming events. We should be able to probe our observation by introducing substituents other than Me on the alkene. As we indicated above, the sequentially of C−O bond forming steps is caused by the transient built up of carbocation character. The introduction of an electron donation substituent, such as OH, should result in stabilisation and emphasise the asynchronous nature of the epoxide formation, whereas the introduction of a substituent with electron withdrawing nature, for example CN, should result in a more concerted reaction. This is indeed the case and can be seen in Figure 3, where the IBO changes associated with the epoxide formation are shown for these substituents. In the presence of the OH groups, the second C−O bond formation trails on along the reaction coordinate, whereas for the CN substituent it is more sudden. For *cis* and *trans* dicyanopropene we also find the epoxide formation to be more concerted in terms of the two forming C−O bonds, as is the case for a single CN substituent. For *trans‐*dicyanopropene in particular, the changes are more drastic. At first glance the epoxide formation becomes more concerted, but a second notable feature is that the proton transfer no longer occurs *after* the epoxide formation but precedes it. The changes in dipole moment, as indicators of hidden ionic intermediates at the proton transfer stages, also reflect this (Figure 4).

Figures 2 and 3 represent quantitative analyses of the variation in electron pair properties as defined by an IBO, but can a further transformation be made into a representation that more closely resembles a curly arrow? The IBOs shown in Figure 2 are contoured at an isosurface value that visually resembles what most people would think of as a bond or a lone pair.

However, the orbitals really are three‐dimensional functions φ(x,y,z), and the two‐dimensional iso‐surface representation is but one of multipe possibilities of condensing important parts of information they carry into a form accessible to human understanding. An even simpler representation, which nevertheless carries information relevant to the curly arrow formalism, is to represent each IBO φ with a single point, its orbital centroid; this is effectively the “average location” of the charge density |φ(**r**)|^2^ represented by the orbital, and is formally defined as:

$$c=\frac{\int |{\varphi \left(r\right)|}^{2}r\;d^{3}r}{\int {|\varphi (r)|}^{2}{\;d}^{3}r}$$

**Figure 5:**
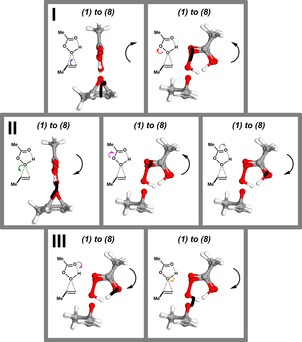
Representation of the IBO changes when reduced to the centroid of the IBOs shown in Figure 3 For every IBO change an approximately in plane orientation is selected. The corresponding “schematic” arrow is shown to the left of the computed arrow, where the black arrow on the right indicates curvature and direction.

So instead of plotting the orbital transformations themselves, one can also reduce each orbital to a characteristic point, and record the coordinates of this point as a minimal representation of the orbital. This approach has been proposed by Vidossich and Lledós.5a, 17 If this is done for all the IBOs computed along an IRC, then one obtains the loci of the complete curve representing that electron pair transformation and hence the curvature of what, with the addition of an arrow head indicating the direction of the reaction, would formally constitute a curly arrow (Figure 5). This reveals that the computed curly arrows bear close resemblance to the schematic arrows as set out in Scheme 1(c), in which the destination of a bond‐forming arrows tends towards the mid‐point of the bond rather than one of the atoms. With polar C−O bonds, the arrow is displaced towards the more electronegative oxygen; with C−C bonds it is likely to be closer to the mid‐point.

### 2.1 Kinetic Isotope Effects

Variation in the substituents on the ethene can be seen to strongly influence whether a proton transfer occurs before or after the C−O bonds are formed. It is of interest therefore to compute the kinetic isotope effects (KIE) resulting from deuterium substitution on both the ethene and on the transferring proton (Table 1). For those transition states where C−O bond formation occurs prior to oxygen to oxygen proton transfer, the KIE is inverse, originating from the change in hybridisation of the carbon carrying a C−H bond from sp^2^ to sp^3^. As the proton transfer starts to anticipate the C−O bond formation, the KIE increases due to a primary effect from the transferring proton. For *cis*‐dicyanopropene, C−O bond formations and proton transfer are approximately synchronous and the KIE reaches a maximum. For *trans*‐dicyanopropene, the transition state occurs after proton transfer, and the primary KIE again decreases. In fact a strong primary KIE is only achieved across a very narrow region; even at this point the effect is less than that expected for a pure proton transfer (∼7) because of the synchronous coupling with the C−O bond formations. To recover these insights from curly arrow representations of the mechanisms does require inclusion of additional information such as that imparted in Figure 3; conventional curly arrows on their own can be insufficient to represent this information.

**Table 1:** Calculated kinetic isotope effects.^[a]^

Ethene substituents	Transition state^b^	ΔG^≠^_298_	Deuterium kinetic isotope effect^c^
Me	10.14469/hpc/3600	29.1	d_3_ on ethene: 0.831 d_4_ on ethane and OH: 0.980
OH	10.14469/hpc/3606	28.9	d_3_ on ethene: 0.820 d_4_ on ethane and OH: 1.093
CN	10.14469/hpc/3602	37.1	d_3_ on ethene: 0.797 d_4_ on ethane and OH: 1.132
cis‐dicyano	10.14469/hpc/4777	40.7	d_3_ on ethane and OH: 2.323
trans‐dicyano	10.14469/hpc/4802	40.2	d_3_ on ethane and OH: 1.139

^a^The overall data collection is available at DOI: 10.14469/hpc/3603. The FAIR data version of this table has DOI: 10.14469/hpc/4931. ^b^Invoke hyperlink to access FAIR data collection for substituent. ^c^At 298 K. KIE data available at DOI: 10.14469/hpc/4812

### 2.2 Computational Details

We computed the reaction pathway at the M06‐2X^18^/Def2‐TZVPPD^19^ level of theory in combination with the CPCM^20^ continuum solvation model for CH_2_Cl_2_ using the electronic structure code Gaussian 16.^21^ Gas phase M06‐2X/Def2‐TZVPPD Kohn‐Sham wave functions were obtained along the IRCs using the electronic structure code ORCA v4.0.1^22^ for IBO analysis, which was carried out using IboView.12a, 15 Calculations in ORCA were accelerated using the RIJCOSX approach^23^ in combination with the AutoAux feature.^24^ Full computational parameters and data can be found in the FAIR data archives.^25^

## 3 Conclusions

In summary, we have demonstrated that the standard textbook mechanism for *e. g*. the closed shell epoxidation of an alkene by a peracid can be placed on a firmer theoretical foundation by deriving and analysing intrinsic bond orbitals (IBOs) along the reaction path. This procedure reveals the importance of including transformations between σ and π bonds. This recognition suggests that the five‐arrow mechanism (Scheme 1) is a better chemical representation of the reaction than the largely ubiquitous four‐arrow variation. Even the former scheme does not consider the σ and π transformations occurring at the carboxylic acid component, and a more complete chemical description of this mechanism benefits from not four or five but seven curly arrows, arranged in three groups. Such grouping or choreography can be used to tease out other features of non‐synchronous reactions such as hidden ionic intermediates and the role of substituents on the alkene or acid.

## Conflict of interest

The authors declare no conflict of interest.

## Supporting information

As a service to our authors and readers, this journal provides supporting information supplied by the authors. Such materials are peer reviewed and may be re‐organized for online delivery, but are not copy‐edited or typeset. Technical support issues arising from supporting information (other than missing files) should be addressed to the authors.

Supplementary
