# Supplementary material for: Epoxidation of Alkenes by Peracids: From Textbook Mechanisms to a Quantum Mechanically Derived Curly‐Arrow Depiction
Source: ChemistryOpen. 2019 Jul 12;8(10):1244–50. doi: 10.1002/open.201900099 (PMC6769425; doi:10.1002/open.201900099)
Supplement: Supplementary file 1 — Supplementary [file OPEN-8-1244-s001.pdf]

Open Access
ChemistryOPEN

Supporting Information

© Copyright Wiley-VCH Verlag GmbH & Co. KGaA, 69451 Weinheim, 2019

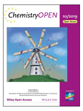

Epoxidation of Alkenes by Peracids: From Textbook Mechanisms to a Quantum Mechanically Derived Curly-Arrow Depiction

Johannes E. M. N. Klein,* Gerald Knizia, and Henry S. Rzepa*© 2019 The Authors. Published by Wiley-VCH Verlag GmbH & Co. KGaA. This is an open access article under the terms of the Creative Commons Attribution License, which permits use, distribution and reproduction in any medium, provided the original work is properly cited.

Table of Contents

1. Data for plots shown in Figure 1	S2-S4
2. Data for plots shown in Figure 2	S4-S6
3. Data for plots shown in Figures 3 and 4	S6-S19

1. Data for plots shown in Figure 1

Table S1: Data for plots shown in Figure 1.

Reaction Coordinate [bohr amu$^{1/2}$]	E [hartree]	ΔE [kcal mol^{-1}][a]	RMS Gradient Norm [hartree bohr^{-1}]	Dipole Moment [debye]
-5.98506	-422.12048	0.36216	4.0714E-5	2.33573
-5.88684	-422.12048	0.36276	3.2348E-5	2.33191
-5.78897	-422.12048	0.3636	4.0047E-5	2.32672
-5.69041	-422.12048	0.3648	3.7827E-5	2.32082
-5.59078	-422.12047	0.36662	2.1183E-5	2.31512
-5.49144	-422.12047	0.36939	4.3466E-5	2.31005
-5.39215	-422.12046	0.37292	5.0189E-5	2.30471
-5.2928	-422.12046	0.37719	5.0849E-5	2.30021
-5.19348	-422.12045	0.38209	5.3418E-5	2.2963
-5.09371	-422.12044	0.38728	2.8758E-5	2.293
-4.99444	-422.12043	0.39277	5.7811E-5	2.29037
-4.89514	-422.12042	0.3982	5.4821E-5	2.28758
-4.79572	-422.12041	0.4041	4.984E-5	2.28559
-4.69615	-422.1204	0.41098	4.2701E-5	2.28461
-4.59636	-422.12039	0.41931	2.5948E-5	2.28466
-4.49663	-422.12037	0.42966	3.9411E-5	2.28567
-4.39679	-422.12035	0.44266	4.0343E-5	2.2877
-4.29688	-422.12032	0.45899	4.5561E-5	2.29027
-4.19695	-422.12029	0.47892	5.2234E-5	2.2937
-4.09699	-422.12026	0.50239	5.5878E-5	2.29775
-3.99704	-422.12021	0.52932	6.5968E-5	2.30236
-3.89708	-422.12016	0.55956	7.2694E-5	2.30741
-3.79713	-422.12011	0.59287	7.8977E-5	2.3128
-3.69717	-422.12005	0.62898	8.4715E-5	2.3182
-3.5972	-422.11999	0.66758	8.7279E-5	2.32339
-3.49725	-422.11993	0.70858	9.4896E-5	2.32848
-3.39731	-422.11986	0.75202	1.00791E-4	2.3333
-3.29738	-422.11978	0.79821	1.07569E-4	2.33771
-3.19746	-422.11971	0.84766	1.15629E-4	2.34193
-3.09754	-422.11962	0.90107	1.21871E-4	2.34592
-2.99764	-422.11953	0.95936	1.37117E-4	2.34977
-2.89774	-422.11943	1.0236	1.50843E-4	2.35333
-2.79784	-422.11931	1.09547	1.68527E-4	2.35702
-2.69794	-422.11918	1.17701	1.91083E-4	2.36093
-2.59801	-422.11903	1.27047	2.1785E-4	2.36542
-2.49807	-422.11886	1.37843	2.53356E-4	2.37053
-2.39812	-422.11866	1.50384	2.94178E-4	2.37703
-2.29817	-422.11843	1.64971	3.41214E-4	2.38544
-2.1982	-422.11816	1.81851	3.93022E-4	2.396
-2.09823	-422.11785	2.01223	4.48857E-4	2.40917
-1.99827	-422.1175	2.23303	5.10359E-4	2.42538
-1.8983	-422.1171	2.48346	5.78184E-4	2.44535
-1.79834	-422.11665	2.76729	6.55674E-4	2.46974
-1.69838	-422.11613	3.08956	7.45325E-4	2.49913
-1.59843	-422.11555	3.45629	8.4873E-4	2.53369
-1.49848	-422.11488	3.87466	9.69826E-4	2.57257
-1.39853	-422.11412	4.35318	0.00111	2.61415
-1.29857	-422.11325	4.90103	0.00127	2.65861
-1.19862	-422.11225	5.5282	0.00145	2.70591
-1.09866	-422.1111	6.24653	0.00167	2.75477
-0.99874	-422.10979	7.07099	0.00192	2.80036
-0.89883	-422.10828	8.02003	0.00222	2.84994
-0.79896	-422.10651	9.13064	0.00263	2.87606
-0.69915	-422.10438	10.46478	0.00321	2.89974
-0.5995	-422.10169	12.15528	0.00426	2.84735
-0.49986	-422.09781	14.58622	0.0064	2.61556
-0.3999	-422.09234	18.01996	0.0083	2.10608
-0.29993	-422.08604	21.9718	0.00855	1.31514
-0.19996	-422.08025	25.60403	0.00693	0.59041
-0.1	-422.07623	28.12761	0.00386	1.66644
0	-422.07478	29.03669	6.225E-6	3.33241
0.1	-422.0763	28.08446	0.00413	5.10402
0.19996	-422.08081	25.25231	0.00811	6.8052
0.29993	-422.08807	20.70224	0.0115	8.27118
0.39991	-422.09744	14.81989	0.01384	9.37126

0.49988	-422.10812	8.12039	0.01505	10.06743
0.59986	-422.11928	1.11212	0.01517	10.40763
0.69983	-422.13016	-5.71347	0.01427	10.46871
0.79981	-422.14004	-11.91487	0.0125	10.32015
0.89975	-422.14838	-17.14432	0.01011	10.02135
0.99962	-422.15487	-21.21915	0.00761	9.62525
1.09927	-422.15972	-24.26276	0.00573	9.1936
1.19893	-422.16353	-26.65294	0.00479	8.75635
1.29869	-422.16695	-28.8015	0.00462	8.31878
1.39857	-422.17041	-30.97282	0.00486	7.83096
1.49851	-422.17415	-33.31626	0.00536	7.26942
1.59843	-422.17836	-35.95597	0.0061	6.61521
1.69838	-422.18308	-38.91906	0.0067	5.93054
1.79834	-422.18801	-42.01499	0.00659	5.26194
1.8983	-422.1925	-44.83299	0.0055	4.66439
1.9982	-422.19595	-46.99397	0.00396	4.20861
2.09793	-422.19849	-48.59271	0.00305	4.01333
2.19776	-422.2005	-49.85473	0.00246	3.92471
2.29762	-422.20215	-50.88865	0.00204	3.88073
2.39753	-422.20353	-51.75313	0.00172	3.84679
2.49744	-422.20469	-52.48383	0.00146	3.83185
2.59735	-422.20569	-53.10624	0.00125	3.81793
2.69727	-422.20654	-53.6403	0.00108	3.81536
2.79719	-422.20728	-54.10371	9.40567E-4	3.81971
2.89712	-422.20793	-54.5112	8.32303E-4	3.83106
2.99707	-422.2085	-54.87407	7.46119E-4	3.84778
3.09702	-422.20903	-55.20169	6.77494E-4	3.86766
3.19699	-422.2095	-55.49984	6.16857E-4	3.89064
3.29696	-422.20993	-55.77051	5.58091E-4	3.91412
3.39694	-422.21032	-56.01461	5.02031E-4	3.93785
3.49692	-422.21067	-56.23425	4.524E-4	3.96123
3.5969	-422.21099	-56.4317	4.05028E-4	3.98377
3.69687	-422.21127	-56.60792	3.60627E-4	4.00534
3.79685	-422.21152	-56.76379	3.17226E-4	4.02444
3.89682	-422.21173	-56.90014	2.76579E-4	4.0419
3.99678	-422.21192	-57.01873	2.41434E-4	4.05885
4.09673	-422.21209	-57.12228	2.09937E-4	4.07606
4.19669	-422.21223	-57.21219	1.82426E-4	4.09485
4.29663	-422.21235	-57.28945	1.57264E-4	4.1154
4.39655	-422.21246	-57.35551	1.3729E-4	4.139
4.49646	-422.21255	-57.41271	1.22142E-4	4.16599
4.59635	-422.21263	-57.46257	1.02571E-4	4.1957
4.69627	-422.2127	-57.50613	9.6642E-5	4.22754
4.79613	-422.21276	-57.54471	8.8362E-5	4.25898
4.89597	-422.21281	-57.57929	8.2673E-5	4.28981
4.99577	-422.21287	-57.61095	8.0727E-5	4.32013
5.09556	-422.21291	-57.64089	6.7048E-5	4.34894
5.19548	-422.21296	-57.66963	7.5527E-5	4.37873
5.29531	-422.213	-57.69759	7.4275E-5	4.4074
5.39513	-422.21305	-57.72481	7.2824E-5	4.43566
5.49496	-422.21309	-57.75126	7.0959E-5	4.46368
5.59479	-422.21313	-57.77693	5.892E-5	4.49014
5.69473	-422.21317	-57.80154	6.8384E-5	4.518
5.79454	-422.21321	-57.82514	6.6058E-5	4.54477
5.89435	-422.21324	-57.84764	6.4201E-5	4.57075
5.99415	-422.21328	-57.86904	6.1957E-5	4.59613
6.09396	-422.21331	-57.88943	4.8416E-5	4.61981
6.19388	-422.21334	-57.90875	6.0146E-5	4.64461
6.29366	-422.21337	-57.92729	5.8334E-5	4.66834
6.39344	-422.2134	-57.94511	5.7232E-5	4.6913
6.49321	-422.21343	-57.96228	5.5487E-5	4.71346
6.593	-422.21345	-57.97888	4.0843E-5	4.73381
6.69292	-422.21348	-57.99473	5.3012E-5	4.75492
6.79269	-422.2135	-58.00995	5.1159E-5	4.77512
6.89246	-422.21352	-58.02446	5.0557E-5	4.79464
6.9922	-422.21355	-58.03822	4.9667E-5	4.8135
7.09193	-422.21357	-58.0512	3.3338E-5	4.83043
7.19185	-422.21359	-58.06313	4.6557E-5	4.84815
7.29157	-422.2136	-58.07405	4.494E-5	4.86517
7.39128	-422.21362	-58.08381	4.3909E-5	4.88127
7.49096	-422.21363	-58.09229	4.2095E-5	4.89657

7.5906	-422.21364	-58.09942	2.324E-5	4.9102
7.69045	-422.21365	-58.10501	3.7761E-5	4.92428
7.78998	-422.21366	-58.10932	3.3769E-5	4.93685
7.88943	-422.21366	-58.11262	3.2145E-5	4.94704
7.98881	-422.21367	-58.11517	3.4196E-5	4.95468
8.08802	-422.21367	-58.11717	1.3424E-5	4.95923

[a]Referenced to -422.121056397 hartree.

2. Data for plots shown in Figure 2

Table S2: Data for plots shown in Figure 2.

Reaction Coordinate [bohr amu$^{1/2}$]	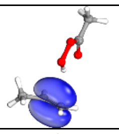	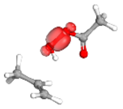	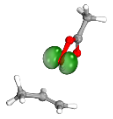	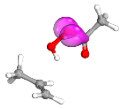	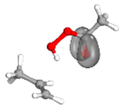	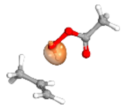	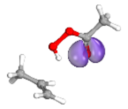
-5.98506	0	0	0	0	0	0	0
-5.88684	1.2E-5	1.4E-5	6E-6	3.4E-5	4.1E-5	8E-6	1.3E-5
-5.78897	4.9E-5	4.7E-5	4E-6	3.9E-5	6.9E-5	4E-6	1.8E-5
-5.69041	1.17E-4	9.1E-5	1.2E-5	8.6E-5	1.38E-4	1.1E-5	4.9E-5
-5.59078	2.03E-4	1.55E-4	1.5E-5	1.18E-4	2E-4	1.5E-5	7.9E-5
-5.49144	2.77E-4	2.2E-4	1.9E-5	1.81E-4	2.77E-4	2.9E-5	1.18E-4
-5.39215	3.63E-4	2.83E-4	2.6E-5	2.11E-4	3.36E-4	3.5E-5	1.51E-4
-5.2928	4.34E-4	3.49E-4	3.2E-5	2.52E-4	4E-4	4.7E-5	1.89E-4
-5.19348	5.05E-4	4.16E-4	3.6E-5	2.89E-4	4.61E-4	6.8E-5	2.31E-4
-5.09371	5.7E-4	4.87E-4	3.9E-5	3.32E-4	5.34E-4	1.05E-4	2.79E-4
-4.99444	6.36E-4	5.56E-4	4.4E-5	3.93E-4	6.08E-4	1.51E-4	3.26E-4
-4.89514	7.45E-4	6.25E-4	5.1E-5	4.35E-4	6.7E-4	2.06E-4	3.75E-4
-4.79572	8.74E-4	6.9E-4	5.9E-5	4.7E-4	7.19E-4	2.71E-4	4.28E-4
-4.69615	0.00102	7.49E-4	6.7E-5	5.02E-4	7.61E-4	3.44E-4	4.87E-4
-4.59636	0.0012	7.98E-4	7.4E-5	5.21E-4	7.89E-4	4.23E-4	5.52E-4
-4.49663	0.00139	8.29E-4	8.1E-5	5.45E-4	8.02E-4	5.12E-4	6.18E-4
-4.39679	0.0016	8.43E-4	8.4E-5	5.46E-4	7.93E-4	5.98E-4	6.88E-4
-4.29688	0.00182	8.45E-4	8.6E-5	5.4E-4	7.69E-4	6.88E-4	7.59E-4
-4.19695	0.00205	8.37E-4	8.6E-5	5.29E-4	7.38E-4	7.81E-4	8.28E-4
-4.09699	0.00229	8.21E-4	8.5E-5	5.11E-4	6.98E-4	8.72E-4	9.01E-4
-3.99704	0.00252	7.9E-4	8.3E-5	4.96E-4	6.49E-4	9.66E-4	9.68E-4
-3.89708	0.00275	7.56E-4	7.9E-5	4.75E-4	5.97E-4	0.00106	0.00104
-3.79713	0.00299	7.13E-4	7.3E-5	4.53E-4	5.41E-4	0.00115	0.00111
-3.69717	0.00321	6.65E-4	6.7E-5	4.24E-4	4.78E-4	0.00125	0.00117
-3.5972	0.00344	6.2E-4	5.8E-5	3.96E-4	4.26E-4	0.00136	0.00122
-3.49725	0.00365	5.63E-4	5.2E-5	3.72E-4	3.66E-4	0.00147	0.00126
-3.39731	0.00385	5E-4	4.9E-5	3.4E-4	3.08E-4	0.00159	0.00129
-3.29738	0.00403	4.25E-4	5.9E-5	3E-4	2.48E-4	0.00171	0.00132
-3.19746	0.00419	3.35E-4	8.7E-5	2.52E-4	1.88E-4	0.00182	0.00134
-3.09754	0.00434	2.24E-4	1.33E-4	1.76E-4	1.36E-4	0.00192	0.00137
-2.99764	0.00446	8.6E-5	1.95E-4	1E-4	1.46E-4	0.002	0.00139
-2.89774	0.00456	1.09E-4	2.68E-4	3.2E-5	2.32E-4	0.00206	0.00141
-2.79784	0.00465	3.01E-4	3.68E-4	1.32E-4	3.58E-4	0.0021	0.00144
-2.69794	0.00472	5.26E-4	4.86E-4	2.7E-4	5.13E-4	0.00212	0.00148
-2.59801	0.00479	7.73E-4	6.18E-4	4.33E-4	6.9E-4	0.00213	0.00153
-2.49807	0.00486	0.00104	7.68E-4	5.98E-4	8.8E-4	0.00212	0.00159
-2.39812	0.00495	0.00133	9.36E-4	7.94E-4	0.0011	0.00211	0.00166
-2.29817	0.00506	0.00164	0.00112	0.00101	0.00135	0.00208	0.00176
-2.1982	0.0052	0.00198	0.00132	0.00124	0.00163	0.00203	0.00188
-2.09823	0.00537	0.00234	0.00153	0.0015	0.00196	0.00197	0.00204
-1.99827	0.00558	0.00276	0.00177	0.00179	0.00234	0.00187	0.00226
-1.8983	0.00583	0.00323	0.00202	0.00213	0.00282	0.00172	0.00254
-1.79834	0.00612	0.00379	0.00229	0.00254	0.00341	0.00149	0.00292
-1.69838	0.00646	0.00448	0.00259	0.00303	0.00415	0.00117	0.00342
-1.59843	0.00683	0.0053	0.00291	0.00361	0.00502	7.46E-4	0.00405
-1.49848	0.00724	0.00629	0.00326	0.00427	0.00605	2.65E-4	0.00481
-1.39853	0.00768	0.00741	0.00363	0.00502	0.0072	5E-4	0.0057
-1.29857	0.00815	0.00869	0.00404	0.00585	0.00848	0.00124	0.00673
-1.19862	0.00866	0.01015	0.00448	0.00677	0.00987	0.00213	0.00791
-1.09866	0.00921	0.01175	0.00497	0.00776	0.0113	0.00319	0.00924
-0.99874	0.00989	0.01379	0.00549	0.00882	0.01278	0.00449	0.01074
-0.89883	0.01058	0.01548	0.00619	0.00972	0.01397	0.00592	0.0124

-0.79896	0.01177	0.01834	0.00695	0.01083	0.01525	0.00773	0.01425
-0.69915	0.01318	0.02087	0.00781	0.01131	0.01577	0.0095	0.01621
-0.5995	0.0165	0.02713	0.009	0.01205	0.01666	0.01145	0.01837
-0.49986	0.02434	0.03977	0.01072	0.01439	0.0193	0.01361	0.02072
-0.3999	0.03961	0.05918	0.01296	0.02031	0.02507	0.01586	0.02317
-0.29993	0.0644	0.08557	0.01654	0.0294	0.03352	0.01809	0.026
-0.19996	0.10206	0.12245	0.0221	0.04155	0.04417	0.02038	0.02933
-0.1	0.15739	0.17674	0.0303	0.05661	0.05756	0.0231	0.03317
0	0.23736	0.25445	0.04166	0.07398	0.07275	0.02676	0.03757
0.1	0.33682	0.34505	0.0565	0.09338	0.08944	0.03169	0.04265
0.19996	0.42834	0.42269	0.07506	0.11395	0.10683	0.03791	0.04836
0.29993	0.49849	0.48014	0.0975	0.13469	0.1241	0.04523	0.05469
0.39991	0.55196	0.5226	0.12415	0.15416	0.14011	0.05323	0.06147
0.49988	0.59758	0.55428	0.1581	0.17107	0.15393	0.06148	0.06843
0.59986	0.65398	0.57727	0.21734	0.18443	0.16484	0.06943	0.0752
0.69983	0.73879	0.59297	0.31355	0.19457	0.17349	0.07715	0.08186
0.79981	0.78567	0.60281	0.36939	0.20205	0.17997	0.08519	0.08859
0.89975	0.80364	0.60888	0.39781	0.2073	0.18506	0.09358	0.09554
0.99962	0.81165	0.61251	0.41777	0.21163	0.18956	0.10256	0.10329
1.09927	0.81561	0.61462	0.43315	0.21516	0.19347	0.11217	0.11251
1.19893	0.81731	0.61591	0.44452	0.22033	0.19897	0.12302	0.12407
1.29869	0.81717	0.61672	0.45184	0.22754	0.20641	0.13582	0.1384
1.39857	0.81566	0.61726	0.45773	0.23504	0.2143	0.15174	0.15633
1.49851	0.81331	0.61759	0.46364	0.24343	0.22318	0.17118	0.17772
1.59843	0.81023	0.61767	0.47001	0.25257	0.233	0.19459	0.20262
1.69838	0.80693	0.61757	0.47631	0.26181	0.24297	0.21938	0.22786
1.79834	0.80364	0.61727	0.48235	0.27069	0.25256	0.24371	0.25161
1.8983	0.80062	0.61679	0.48782	0.27862	0.26118	0.26598	0.27241
1.9982	0.79816	0.61611	0.49226	0.2846	0.26783	0.28449	0.2887
2.09793	0.79678	0.61537	0.4947	0.28686	0.27076	0.29567	0.29844
2.19776	0.79594	0.61476	0.49607	0.28786	0.27251	0.30302	0.30473
2.29762	0.79535	0.61425	0.497	0.28842	0.27385	0.30844	0.30926
2.39753	0.79487	0.61379	0.49775	0.28903	0.27522	0.31301	0.31287
2.49744	0.79452	0.6134	0.49827	0.28944	0.27634	0.31656	0.3156
2.59735	0.79425	0.61305	0.49868	0.28987	0.27743	0.31966	0.31782
2.69727	0.79408	0.61275	0.49894	0.29014	0.27826	0.32214	0.3195
2.79719	0.79398	0.61252	0.49907	0.29031	0.27891	0.3242	0.32077
2.89712	0.79395	0.61235	0.49911	0.29039	0.27938	0.32588	0.3217
2.99707	0.79397	0.61223	0.49906	0.2904	0.2797	0.32727	0.32238
3.09702	0.79402	0.61215	0.49897	0.29037	0.27992	0.32849	0.32289
3.19699	0.7941	0.61211	0.49885	0.29029	0.28004	0.32958	0.32328
3.29696	0.79422	0.61207	0.4986	0.28951	0.27958	0.33062	0.32379
3.39694	0.7943	0.61205	0.49848	0.28941	0.27964	0.33162	0.32411
3.49692	0.79437	0.61204	0.49837	0.28933	0.27968	0.33262	0.32443
3.5969	0.79443	0.61203	0.49829	0.28926	0.27974	0.33365	0.32478
3.69687	0.79444	0.61204	0.49836	0.28985	0.28031	0.33469	0.32499
3.79685	0.79447	0.61203	0.49832	0.28983	0.28039	0.33579	0.32541
3.89682	0.79449	0.61201	0.49831	0.28983	0.28049	0.33691	0.32588
3.99678	0.7945	0.61199	0.49832	0.28985	0.28061	0.33803	0.32638
4.09673	0.79449	0.61197	0.49835	0.28991	0.28074	0.33914	0.3269
4.19669	0.79448	0.61196	0.49838	0.28995	0.28087	0.34016	0.3274
4.29663	0.79446	0.61194	0.49841	0.28998	0.28098	0.34109	0.32786
4.39655	0.79445	0.61192	0.49843	0.29	0.28107	0.34189	0.32824
4.49646	0.79444	0.6119	0.49844	0.29	0.28114	0.34255	0.32856
4.59635	0.79444	0.61189	0.49845	0.29	0.28119	0.3431	0.32883
4.69627	0.79444	0.61188	0.49844	0.28999	0.28124	0.3435	0.32904
4.79613	0.79443	0.61187	0.49844	0.29	0.28129	0.34381	0.32922
4.89597	0.7944	0.61186	0.49845	0.29003	0.28134	0.34396	0.32938
4.99577	0.79438	0.61185	0.49846	0.29005	0.28137	0.34393	0.32946
5.09556	0.79434	0.61185	0.49848	0.29007	0.28138	0.3437	0.32948
5.19548	0.79432	0.61185	0.49848	0.29008	0.28138	0.34331	0.32942
5.29531	0.7943	0.61186	0.49847	0.29007	0.28136	0.34284	0.32932
5.39513	0.79429	0.61186	0.49846	0.29006	0.28133	0.34231	0.32919
5.49496	0.79428	0.61187	0.49844	0.29005	0.2813	0.34175	0.32905
5.59479	0.79427	0.61187	0.49842	0.29004	0.28126	0.34119	0.32891
5.69473	0.79427	0.61188	0.49838	0.29002	0.28123	0.34062	0.32876
5.79454	0.79427	0.61189	0.49835	0.28999	0.28119	0.34007	0.32861
5.89435	0.79427	0.61189	0.49832	0.28998	0.28117	0.33955	0.32849
5.99415	0.79427	0.6119	0.4983	0.28996	0.28114	0.33906	0.32837
6.09396	0.79426	0.6119	0.49829	0.28997	0.28114	0.33865	0.32831
6.19388	0.79426	0.6119	0.49826	0.28996	0.28113	0.33825	0.32824

6.29366	0.79425	0.61191	0.49824	0.28996	0.28113	0.33789	0.32818
6.39344	0.79424	0.61191	0.49823	0.28997	0.28114	0.33759	0.32816
6.49321	0.79423	0.61191	0.49823	0.28999	0.28116	0.33735	0.32817
6.593	0.79422	0.61191	0.49824	0.29002	0.28118	0.33717	0.32821
6.69292	0.79421	0.6119	0.49823	0.29004	0.28121	0.33695	0.32823
6.79269	0.79421	0.6119	0.49822	0.29006	0.28123	0.3367	0.32823
6.89246	0.79424	0.61188	0.49809	0.28941	0.28073	0.33643	0.32838
6.9922	0.79424	0.61188	0.49807	0.28942	0.28074	0.33614	0.32836
7.09193	0.79424	0.61188	0.49806	0.28944	0.28075	0.33586	0.32835
7.19185	0.7942	0.6119	0.49816	0.29015	0.28129	0.33554	0.32815
7.29157	0.7942	0.61189	0.49813	0.29016	0.2813	0.33522	0.3281
7.39128	0.7942	0.61189	0.49809	0.29016	0.2813	0.33492	0.32806
7.49096	0.79425	0.61187	0.49793	0.28945	0.28077	0.33463	0.32819
7.5906	0.79424	0.61187	0.4979	0.28946	0.28079	0.33441	0.32819
7.69045	0.7942	0.61188	0.498	0.29018	0.28134	0.3342	0.32801
7.78998	0.79424	0.61186	0.49784	0.28947	0.28082	0.33402	0.32816
7.88943	0.79424	0.61186	0.49781	0.28948	0.28083	0.3339	0.32816
7.98881	0.79424	0.61186	0.49779	0.28948	0.28085	0.3338	0.32817
8.08802	0.79419	0.61187	0.49791	0.29023	0.28141	0.33376	0.32804

3. Data for plots shown in Figures 3 and 4

Table S3: Data shown in Figures 3 and 4 for *trans* dicyanopropene.

Reaction Coordinate [bohr amu$^{1/2}$]	E [hartree]	ΔE [kcal mol^{-1}][a]	IBO Changes for π-Bond Olefin	IBO Changes for Lone Pair on Oxygen	Dipole Moment [debye]
-20.1808	-606.62532	0.67136	0	0	3.75643
-20.00126	-606.62531	0.67551	8.9E-5	8E-5	3.75182
-19.82546	-606.62531	0.67813	2.83E-4	4.3E-5	3.77233
-19.64596	-606.6253	0.68578	1.74E-4	3.57E-4	3.77259
-19.477	-606.62527	0.70105	0.00119	8E-5	3.78863
-19.30058	-606.62525	0.71221	2.8E-4	3.06E-4	3.81892
-19.12039	-606.62526	0.71049	8.4E-4	3.8E-4	3.83557
-18.93907	-606.62526	0.71051	9.87E-4	1.76E-4	3.84761
-18.75717	-606.62524	0.71898	0.00145	4.55E-4	3.87877
-18.56684	-606.62525	0.71418	0.00158	3.83E-4	3.88544
-18.37	-606.62524	0.72332	0.00206	4.85E-4	3.89992
-18.17119	-606.62521	0.74266	0.00252	5.4E-4	3.90885
-17.97218	-606.62516	0.76936	0.00301	5.94E-4	3.92672
-17.77346	-606.62512	0.7982	0.00346	6.27E-4	3.9433
-17.57431	-606.62508	0.82487	0.00395	6.45E-4	3.9612
-17.38237	-606.62502	0.85675	0.00442	6.45E-4	3.93957
-17.18854	-606.62499	0.87762	0.0046	6.74E-4	3.98683
-16.99053	-606.62494	0.90697	0.00517	6.6E-4	3.96819
-16.79245	-606.62488	0.94466	0.00527	7.2E-4	3.99091
-16.59322	-606.62482	0.98403	0.00589	7.15E-4	3.99181
-16.39482	-606.62476	1.0254	0.00605	7.32E-4	4.01532
-16.19546	-606.62469	1.06466	0.00637	6.92E-4	4.02078
-15.99645	-606.62463	1.10318	0.00647	6.79E-4	4.02555
-15.79689	-606.62457	1.14223	0.007	6.36E-4	4.02782
-15.59739	-606.62451	1.17945	0.00707	6.13E-4	4.03014
-15.40359	-606.62444	1.2225	0.00764	5.22E-4	4.02376
-15.21078	-606.62438	1.26036	0.00752	9.92E-4	4.03883
-15.01591	-606.62433	1.29149	0.00808	5.2E-4	4.03372
-14.82068	-606.62429	1.3203	0.00789	9.4E-4	4.04638
-14.62256	-606.62424	1.34622	0.00842	9.46E-4	4.04852
-14.42538	-606.6242	1.37511	0.00826	9.02E-4	4.05798
-14.22675	-606.62416	1.3987	0.00864	9.12E-4	4.0636
-14.02902	-606.62413	1.42086	0.00882	8.57E-4	4.06762
-13.83053	-606.6241	1.43932	0.00896	8.58E-4	4.07166
-13.63209	-606.62407	1.45595	0.00913	8.35E-4	4.0743
-13.44199	-606.62403	1.48219	0.00948	6.25E-4	4.066
-13.24804	-606.62402	1.48932	0.00943	7.96E-4	4.08656
-13.05085	-606.62399	1.5031	0.00967	8.27E-4	4.07625
-12.85329	-606.62397	1.51917	0.00965	5.7E-4	4.08573
-12.65539	-606.62394	1.53628	0.00988	5.86E-4	4.08059
-12.45754	-606.62391	1.55454	0.01002	5.5E-4	4.08776
-12.26043	-606.62389	1.57133	0.01014	5.1E-4	4.07775
-12.06434	-606.62386	1.58532	0.01033	5.33E-4	4.08971

-11.86775	-606.62384	1.59699	0.01031	4.99E-4	4.07892
-11.67326	-606.62383	1.60889	0.01057	5.63E-4	4.09001
-11.49356	-606.62377	1.64268	0.01055	4.45E-4	4.06247
-11.30793	-606.62377	1.64233	0.01051	5.41E-4	4.09086
-11.12094	-606.62376	1.65173	0.01065	7.34E-4	4.05459
-10.93174	-606.62374	1.66254	0.01067	4.85E-4	4.06901
-10.73816	-606.62373	1.66817	0.01063	4.92E-4	4.05573
-10.54733	-606.62371	1.68065	0.01093	5.11E-4	4.05163
-10.35858	-606.62368	1.6978	0.01054	6.4E-4	4.05038
-10.17149	-606.62366	1.71082	0.01087	5.47E-4	4.04106
-9.98352	-606.62364	1.7244	0.01037	6.74E-4	4.03961
-9.79199	-606.62363	1.73486	0.01072	5.96E-4	4.03049
-9.60809	-606.62357	1.76881	0.01022	5.55E-4	4.03995
-9.42739	-606.62354	1.78594	0.01045	5.64E-4	4.0007
-9.24497	-606.62353	1.79741	0.01039	5.48E-4	4.01147
-9.06076	-606.62351	1.80805	0.01046	6.56E-4	3.97125
-8.87577	-606.62348	1.82624	0.01062	6.48E-4	3.97977
-8.68537	-606.62346	1.83921	0.01047	6.05E-4	3.93123
-8.48879	-606.62345	1.84588	0.01068	6.3E-4	3.92972
-8.28989	-606.62341	1.86735	0.01057	5.95E-4	3.90127
-8.09186	-606.62338	1.8915	0.01057	7.1E-4	3.88102
-7.89297	-606.62334	1.91484	0.01034	6.27E-4	3.8627
-7.7039	-606.62328	1.95055	0.0103	5.15E-4	3.83382
-7.51697	-606.62326	1.96577	0.01001	5.71E-4	3.8351
-7.32731	-606.62325	1.97248	0.01004	5.75E-4	3.81018
-7.13582	-606.62323	1.98265	0.00994	5.62E-4	3.78742
-6.94559	-606.6232	2.00229	0.01002	5.06E-4	3.76782
-6.76058	-606.62314	2.0366	0.00972	4.68E-4	3.72735
-6.5719	-606.62315	2.03595	0.00989	5.32E-4	3.72774
-6.38208	-606.62313	2.04285	0.00939	4.62E-4	3.69358
-6.19867	-606.62308	2.07666	0.00878	5.27E-4	3.69173
-6.02301	-606.62303	2.10603	0.00934	4.81E-4	3.63584
-5.83994	-606.62297	2.14874	0.00806	4.55E-4	3.65048
-5.65955	-606.6229	2.19039	0.00857	4.02E-4	3.62062
-5.46895	-606.62295	2.16024	0.00765	3.75E-4	3.60954
-5.27989	-606.6229	2.18814	0.00792	3.78E-4	3.59221
-5.09489	-606.62283	2.23333	0.00754	3.48E-4	3.56247
-4.90226	-606.62281	2.24517	0.00743	3.42E-4	3.55401
-4.706	-606.62277	2.27043	0.00755	3.35E-4	3.51299
-4.50864	-606.62271	2.30941	0.00748	3.54E-4	3.48168
-4.31199	-606.62262	2.36362	0.0076	4.33E-4	3.44468
-4.11287	-606.62251	2.43684	0.00774	5.26E-4	3.40628
-3.91412	-606.62234	2.54266	0.00816	5.74E-4	3.35954
-3.71515	-606.62211	2.68508	0.00843	8.93E-4	3.31106
-3.51568	-606.6218	2.87963	0.00879	0.00122	3.26175
-3.31752	-606.62139	3.1387	0.00897	0.00185	3.22911
-3.12014	-606.62086	3.46751	0.00926	0.00219	3.23058
-2.94401	-606.61947	4.34537	0.00942	0.00313	3.29706
-2.76376	-606.61855	4.92048	0.00928	0.00307	3.45137
-2.56957	-606.61768	5.46266	0.00925	0.00405	3.72775
-2.37281	-606.6151	7.08387	0.00911	0.00453	4.01225
-2.17412	-606.61069	9.84939	0.00911	0.00618	4.42933
-1.97565	-606.60385	14.14102	0.00937	0.00964	4.97886
-1.77946	-606.59799	17.82175	0.01023	0.01542	5.5609
-1.58351	-606.59427	20.15629	0.01159	0.02027	6.00015
-1.38553	-606.59206	21.54406	0.01281	0.02605	6.37526
-1.18694	-606.59021	22.70599	0.01498	0.0288	6.63147
-0.98978	-606.58825	23.93594	0.01691	0.03471	6.87655
-0.79477	-606.5858	25.46857	0.02071	0.03327	7.06364
-0.59931	-606.58266	27.44075	0.02216	0.04342	7.33184
-0.39989	-606.578	30.36436	0.03408	0.04362	7.44815
-0.2	-606.5694	35.76119	0.07127	0.05537	6.81691
7E-5	-606.56423	39.00338	0.16599	0.09104	5.27237
0.2	-606.57131	34.56557	0.30951	0.14429	3.40565
0.39993	-606.59168	21.77853	0.43957	0.21433	2.58915
0.59986	-606.61973	4.1811	0.69649	0.46812	2.65452
0.7998	-606.64821	-13.69311	0.74068	0.50953	2.63214
0.99973	-606.67018	-27.48187	0.7506	0.52002	2.45674
1.19874	-606.67959	-33.38131	0.75604	0.52422	2.30778
1.39317	-606.68304	-35.54874	0.75687	0.5233	2.37007
1.59109	-606.68472	-36.60035	0.75632	0.5249	2.41513

1.78239	-606.68632	-37.60967	0.75846	0.52206	2.51014
1.97063	-606.68817	-38.76603	0.7582	0.52353	2.46069
2.16676	-606.68962	-39.6768	0.75867	0.52268	2.4993
2.36667	-606.69081	-40.42677	0.75911	0.52217	2.51158
2.56655	-606.69184	-41.07343	0.75956	0.52169	2.52008
2.76643	-606.69274	-41.63717	0.76006	0.52118	2.53027
2.96632	-606.69353	-42.12817	0.76057	0.52067	2.54195
3.16621	-606.69421	-42.55897	0.76104	0.52019	2.5545
3.36608	-606.6948	-42.93021	0.76146	0.51975	2.56703
3.56593	-606.69529	-43.23366	0.76182	0.51937	2.58069
3.76567	-606.69567	-43.47426	0.76209	0.51909	2.59371
3.96543	-606.69598	-43.66561	0.76241	0.51877	2.60826
4.16474	-606.69622	-43.81828	0.76281	0.51844	2.62483
4.36455	-606.69641	-43.93786	0.76309	0.51823	2.64481
4.56388	-606.69656	-44.03009	0.76331	0.5181	2.67088
4.76279	-606.69665	-44.08981	0.76345	0.51803	2.68814
4.95958	-606.69671	-44.12798	0.76346	0.51801	2.69063
5.15645	-606.69676	-44.1609	0.76355	0.51788	2.6873
5.3539	-606.69681	-44.18701	0.76357	0.51782	2.68911
5.54889	-606.69679	-44.17455	0.76358	0.51773	2.68283
5.73165	-606.6968	-44.18619	0.76357	0.51769	2.66923
5.91147	-606.69685	-44.21243	0.7637	0.51753	2.70183
6.09091	-606.69693	-44.26409	0.7637	0.51741	2.66951
6.28765	-606.69696	-44.28383	0.7636	0.51743	2.64583
6.48409	-606.697	-44.31002	0.76365	0.51723	2.62089
6.68248	-606.69705	-44.33836	0.76364	0.51713	2.59229
6.87889	-606.69709	-44.36671	0.76359	0.51708	2.56635
7.07483	-606.69711	-44.37963	0.76352	0.51706	2.54222
7.26315	-606.69714	-44.39781	0.76352	0.51689	2.51447
7.45078	-606.69709	-44.36449	0.76349	0.51692	2.49261
7.63105	-606.69717	-44.41378	0.76348	0.51696	2.49877
7.8085	-606.69727	-44.47668	0.76347	0.51669	2.44332
7.99423	-606.69734	-44.52461	0.76349	0.51654	2.42382
8.19028	-606.6974	-44.56157	0.76347	0.51641	2.38191
8.38725	-606.69745	-44.59154	0.76344	0.51623	2.34329
8.5808	-606.69747	-44.60572	0.76339	0.51617	2.30997
8.76877	-606.69754	-44.6472	0.76335	0.51603	2.29349
8.95512	-606.69765	-44.71477	0.76332	0.51591	2.23791
9.14626	-606.69773	-44.76438	0.76331	0.51574	2.22334
9.33524	-606.69783	-44.83157	0.7633	0.51563	2.16326
9.52606	-606.69791	-44.87963	0.7633	0.51543	2.16702
9.71373	-606.69804	-44.96093	0.76332	0.51532	2.10221
9.9052	-606.69818	-45.04797	0.7633	0.51513	2.09472
10.10355	-606.69828	-45.10993	0.76332	0.51493	2.07433
10.30209	-606.69836	-45.162	0.76335	0.51463	2.06031
10.49945	-606.69841	-45.19086	0.76334	0.51457	2.06266
10.6927	-606.69842	-45.19974	0.76331	0.51437	2.06073
10.87792	-606.69847	-45.23067	0.76335	0.51435	2.06107
11.0651	-606.69852	-45.26028	0.76325	0.51446	2.09079
11.25479	-606.69853	-45.2677	0.76335	0.51426	2.07473
11.44328	-606.6985	-45.25232	0.76338	0.51424	2.09232
11.62406	-606.69857	-45.295	0.76323	0.51448	2.11372
11.80802	-606.69863	-45.32885	0.76331	0.5144	2.11763
12.00736	-606.69866	-45.34922	0.76329	0.51444	2.12914
12.20624	-606.69869	-45.3714	0.76326	0.51447	2.1396
12.40509	-606.69873	-45.39448	0.76325	0.51447	2.14727
12.60379	-606.69877	-45.41841	0.76325	0.51445	2.15081
12.80221	-606.69881	-45.44423	0.76326	0.51441	2.15371
13.00026	-606.69885	-45.47108	0.7633	0.51432	2.15056
13.19479	-606.69887	-45.4833	0.7633	0.51429	2.16398
13.38267	-606.69881	-45.44706	0.76333	0.51425	2.1461
13.5626	-606.69881	-45.44648	0.76341	0.51412	2.13931
13.73237	-606.699	-45.56512	0.76342	0.51398	2.13738
13.92119	-606.69904	-45.58872	0.76348	0.51415	2.15998
14.10872	-606.69909	-45.62111	0.76343	0.51412	2.15975
14.30066	-606.69913	-45.64656	0.76347	0.51416	2.16247
14.4926	-606.69916	-45.66521	0.76346	0.51414	2.16802
14.68183	-606.69918	-45.67812	0.76352	0.51414	2.17147
14.86875	-606.6992	-45.68878	0.76347	0.51417	2.17768
15.05367	-606.6992	-45.69175	0.76363	0.51396	2.19453
15.23702	-606.69922	-45.69872	0.76359	0.51402	2.1839

15.41871	-606.69921	-45.69277	0.76367	0.51381	2.17751
15.58854	-606.69928	-45.7414	0.76366	0.51386	2.17635
15.77283	-606.69928	-45.74192	0.76371	0.51387	2.19834
15.95077	-606.69929	-45.74678	0.76371	0.51377	2.13766
16.13318	-606.6993	-45.74883	0.7638	0.51362	2.14525
16.31635	-606.6993	-45.74892	0.76375	0.51369	2.09941
16.49914	-606.69926	-45.72636	0.76384	0.51347	2.11296
16.67875	-606.69922	-45.70141	0.76388	0.51362	2.06986
16.8546	-606.69925	-45.71747	0.76385	0.51354	2.08609
17.03071	-606.69927	-45.73591	0.76392	0.51351	2.0446
17.21184	-606.69926	-45.72757	0.76399	0.51333	2.02705
17.38432	-606.69932	-45.76141	0.76399	0.51341	2.02364
17.58077	-606.69932	-45.76614	0.76406	0.51326	2.01054
17.77621	-606.69933	-45.76818	0.7641	0.51332	2.01775

[a]Referenced to -606.626389823 hartree.

Table S4: Data shown in Figures 3 and 4 for *cis* dicyanopropene.

Reaction Coordinate [bohr amu$^{1/2}$]	E [hartree]	ΔE [kcal mol^{-1}][a]	IBO Changes for π-Bond Olefin	IBO Changes for Lone Pair on Oxygen	Dipole Moment [debye]
-16.09258	-606.62463	0.51047	0	0	10.5509
-15.89501	-606.62463	0.51056	4.4E-5	6.4E-5	10.55121
-15.70956	-606.62463	0.51022	6.3E-5	4.9E-5	10.55089
-15.51574	-606.62462	0.51363	3.1E-4	5.7E-5	10.55334
-15.33522	-606.62459	0.53827	9.49E-4	5.9E-5	10.57581
-15.15635	-606.62459	0.53841	5.52E-4	7.5E-5	10.53643
-14.97923	-606.62458	0.54129	6.2E-5	4.4E-5	10.55825
-14.8011	-606.62459	0.53651	2.14E-4	8.9E-5	10.5511
-14.61149	-606.6246	0.53001	8.94E-4	9.1E-5	10.54518
-14.4137	-606.62458	0.54238	0.00115	1.46E-4	10.53825
-14.21634	-606.62454	0.56445	0.00174	1.48E-4	10.52973
-14.01901	-606.62451	0.58829	0.00214	1.92E-4	10.51416
-13.82998	-606.62444	0.62695	0.00248	1.79E-4	10.4966
-13.64808	-606.62439	0.66185	0.00375	2.49E-4	10.47971
-13.46884	-606.62429	0.72167	0.00299	1.63E-4	10.52526
-13.28508	-606.6243	0.71876	0.00424	3.56E-4	10.44902
-13.09617	-606.62427	0.73535	0.00417	3.25E-4	10.46469
-12.90627	-606.62421	0.77436	0.00495	3.57E-4	10.44376
-12.71418	-606.62414	0.81565	0.00521	3.92E-4	10.42202
-12.52126	-606.62408	0.85399	0.00585	4.31E-4	10.41577
-12.32342	-606.62403	0.88569	0.00594	4.34E-4	10.38934
-12.1256	-606.62397	0.92632	0.00638	4.41E-4	10.37048
-11.9262	-606.6239	0.96867	0.00667	4.61E-4	10.34467
-11.72737	-606.62383	1.01253	0.00713	4.7E-4	10.32342
-11.53958	-606.62373	1.07496	0.00741	5E-4	10.28419
-11.35146	-606.62368	1.10961	0.00779	4.99E-4	10.28868
-11.15541	-606.62364	1.13087	0.00805	5.3E-4	10.24925
-10.95727	-606.62358	1.16672	0.00839	5.33E-4	10.22543
-10.75797	-606.62352	1.20758	0.00864	5.33E-4	10.19211
-10.55882	-606.62345	1.24926	0.00885	5.41E-4	10.16188
-10.35936	-606.62339	1.29121	0.00906	5.21E-4	10.1287
-10.15986	-606.62332	1.33321	0.0092	5.08E-4	10.09526
-9.96039	-606.62326	1.37319	0.00933	4.69E-4	10.0615
-9.76082	-606.6232	1.40958	0.00939	4.53E-4	10.02742
-9.5674	-606.62313	1.44858	0.00961	4.8E-4	9.97865
-9.37874	-606.62308	1.48224	0.00933	4.27E-4	9.97712
-9.18973	-606.62304	1.50899	0.00976	4.83E-4	9.91756
-9.00002	-606.623	1.53157	0.00935	4.68E-4	9.91509
-8.81012	-606.62296	1.55568	0.0098	4.58E-4	9.86804
-8.62171	-606.62292	1.58521	0.00949	4.49E-4	9.84212
-8.4307	-606.62289	1.59984	0.01005	4.54E-4	9.80016
-8.23352	-606.62288	1.61119	0.00986	4.3E-4	9.76553
-8.03601	-606.62284	1.63183	0.01002	4.21E-4	9.72329
-7.83718	-606.62281	1.65427	0.00999	4.19E-4	9.68155
-7.64777	-606.62275	1.6893	0.00994	4.02E-4	9.64108
-7.4588	-606.62272	1.71169	0.01005	4.32E-4	9.6107
-7.27344	-606.62266	1.74473	0.01006	4.07E-4	9.57363
-7.09516	-606.62258	1.79969	0.00998	4.69E-4	9.52887
-6.91414	-606.62257	1.80507	0.00964	2.69E-4	9.53068
-6.72861	-606.62259	1.79364	0.00989	4.53E-4	9.47216

-6.54714	-606.62252	1.83585	0.00918	2.33E-4	9.46068
-6.35922	-606.62253	1.82606	0.01018	4.32E-4	9.40177
-6.16326	-606.62251	1.84139	0.00948	4.1E-4	9.38626
-5.96787	-606.62246	1.8694	0.00965	4.02E-4	9.34964
-5.78088	-606.62238	1.92367	0.00904	6.29E-4	9.3434
-5.59458	-606.62236	1.93423	0.00911	3.71E-4	9.31138
-5.4104	-606.62231	1.96872	0.00873	4.43E-4	9.29919
-5.23349	-606.62221	2.02608	0.00838	3.68E-4	9.2928
-5.05565	-606.62219	2.03977	0.00857	4.63E-4	9.2602
-4.87488	-606.62219	2.04186	0.00748	2.97E-4	9.25675
-4.69346	-606.62218	2.04507	0.00821	4.03E-4	9.22811
-4.50298	-606.6222	2.03756	0.00719	4.02E-4	9.21964
-4.30885	-606.62217	2.05391	0.00702	3.85E-4	9.20892
-4.11044	-606.62213	2.07665	0.00635	3.78E-4	9.20633
-3.93393	-606.62199	2.16516	0.00577	3.4E-4	9.20669
-3.73812	-606.62205	2.13118	0.00526	3.64E-4	9.20729
-3.54656	-606.62201	2.15253	0.00502	4.38E-4	9.19735
-3.35008	-606.62195	2.19203	0.00492	5.84E-4	9.17992
-3.15122	-606.62183	2.26484	0.00477	8.02E-4	9.16205
-2.95169	-606.62165	2.38036	0.00474	0.00108	9.14269
-2.75204	-606.62139	2.54529	0.00472	0.00142	9.12201
-2.55234	-606.62103	2.7694	0.00463	0.00182	9.10205
-2.3528	-606.62056	3.06643	0.00457	0.0023	9.08958
-2.15323	-606.61993	3.4576	0.00438	0.00294	9.09092
-1.96013	-606.61901	4.03388	0.00457	0.00356	9.12467
-1.77211	-606.61775	4.82789	0.00469	0.00455	9.17269
-1.58428	-606.61608	5.87638	0.00512	0.00564	9.28262
-1.3899	-606.61446	6.88989	0.00605	0.00655	9.30188
-1.19277	-606.61175	8.59426	0.00653	0.00893	9.50738
-0.99688	-606.60786	11.03125	0.00964	0.01029	9.64147
-0.79867	-606.60301	14.07675	0.00918	0.01441	9.93493
-0.5998	-606.59583	18.5853	0.01826	0.01877	10.09008
-0.39991	-606.58206	27.22414	0.04823	0.0271	9.67124
-0.2	-606.56723	36.53159	0.12202	0.04661	8.68699
9E-5	-606.56096	40.46577	0.27834	0.08252	7.72514
0.2	-606.56773	36.21581	0.44976	0.13446	7.69452
0.39992	-606.58608	24.70375	0.57164	0.22277	8.23534
0.59984	-606.6097	9.88052	0.74442	0.4149	8.40075
0.79974	-606.63259	-4.48533	0.76953	0.46218	8.23746
0.9994	-606.65201	-16.66889	0.77152	0.48919	8.12331
1.19903	-606.66687	-25.99545	0.76855	0.5063	8.27224
1.39811	-606.67369	-30.278	0.76564	0.51515	8.54025
1.59243	-606.67797	-32.96086	0.76653	0.51532	8.58029
1.78633	-606.68104	-34.88635	0.76546	0.51357	8.7827
1.97835	-606.68395	-36.7128	0.76478	0.51829	8.8723
2.17338	-606.68622	-38.14091	0.76437	0.51818	8.94009
2.37297	-606.68793	-39.21018	0.76412	0.51861	8.97684
2.57268	-606.68932	-40.08603	0.76401	0.51887	8.99907
2.77252	-606.69049	-40.81809	0.76401	0.51897	9.01236
2.97241	-606.69148	-41.43745	0.76406	0.51896	9.02173
3.17233	-606.69233	-41.96991	0.76414	0.51888	9.03154
3.37225	-606.69307	-42.43377	0.76424	0.51879	9.04657
3.57217	-606.6937	-42.83448	0.76426	0.51885	9.06619
3.77209	-606.69425	-43.1751	0.7643	0.51883	9.08979
3.972	-606.6947	-43.45721	0.76437	0.51878	9.11409
4.17185	-606.69506	-43.68421	0.76445	0.51868	9.13677
4.37175	-606.69534	-43.86132	0.76458	0.51853	9.16047
4.5715	-606.69556	-43.99911	0.76462	0.51852	9.18402
4.7711	-606.69573	-44.10839	0.7647	0.51845	9.20765
4.9706	-606.69587	-44.19296	0.76483	0.51834	9.23126
5.16963	-606.69596	-44.25017	0.7649	0.51822	9.2492
5.3674	-606.69602	-44.28661	0.76494	0.51809	9.26207
5.56404	-606.69606	-44.31023	0.76494	0.51802	9.26813
5.75863	-606.69609	-44.32877	0.76496	0.51802	9.275
5.94922	-606.69608	-44.32401	0.76493	0.51795	9.25453
6.13656	-606.69617	-44.38138	0.76498	0.51793	9.25171
6.33606	-606.6962	-44.40345	0.76495	0.51791	9.24125
6.53376	-606.69623	-44.42228	0.7649	0.51788	9.22497
6.73207	-606.69626	-44.43833	0.76485	0.51788	9.20484
6.93023	-606.69628	-44.45231	0.76484	0.51782	9.18622
7.12728	-606.69629	-44.45949	0.76472	0.518	9.16541

7.3194	-606.69629	-44.45747	0.76488	0.51768	9.16619
7.50191	-606.69627	-44.44497	0.76475	0.51799	9.12506
7.68269	-606.69614	-44.3628	0.76454	0.5178	9.14604
7.86031	-606.69615	-44.36711	0.7648	0.51776	9.09629
8.03246	-606.69632	-44.47582	0.7647	0.51787	9.07723
8.23064	-606.69632	-44.47752	0.76474	0.5177	9.06365

[a]Referenced to -606.625443349 hartree.

Table S5: Data shown in Figures 3 and 4 for cyanoethene.

Reaction Coordinate [bohr amu$^{1/2}$]	E [hartree]	ΔE [kcal mol^{-1}][a]	IBO Changes for π-Bond Olefin	IBO Changes for Lone Pair on Oxygen	Dipole Moment [debye]
-9.77059	-475.06128	-0.00412	0	0	7.24273
-9.67136	-475.06128	-0.00306	2.9E-5	1E-6	7.24235
-9.57241	-475.06128	-0.00155	5.7E-5	4.54E-4	7.24195
-9.47435	-475.06127	-2.9493E-5	1.2E-4	4.02E-4	7.23818
-9.37568	-475.06127	0.00193	1.85E-4	3.51E-4	7.2342
-9.2767	-475.06127	0.00452	2.68E-4	0.00184	7.2276
-9.1776	-475.06126	0.00794	3.7E-4	0.00189	7.21922
-9.07793	-475.06125	0.01221	4.69E-4	0.00196	7.20869
-8.97887	-475.06125	0.0177	5.49E-4	0.00202	7.19793
-8.87956	-475.06124	0.02389	6.44E-4	0.00208	7.18355
-8.78016	-475.06122	0.031	7.2E-4	0.00213	7.16724
-8.68067	-475.06121	0.03904	7.85E-4	0.00217	7.15071
-8.58121	-475.0612	0.04763	8.65E-4	0.0022	7.13426
-8.4814	-475.06118	0.05661	9.49E-4	0.00223	7.11726
-8.38195	-475.06117	0.06646	0.00103	0.00225	7.10083
-8.28243	-475.06115	0.0768	0.00112	0.00243	7.08273
-8.18285	-475.06113	0.08797	0.00121	0.00244	7.06491
-8.08326	-475.06111	0.10018	0.0013	0.00246	7.04785
-7.98374	-475.06109	0.11349	0.00137	0.00247	7.03079
-7.8839	-475.06107	0.12755	0.00143	0.00248	7.01273
-7.78435	-475.06105	0.14235	0.00147	0.00223	6.99507
-7.68476	-475.06102	0.15728	0.00152	0.00226	6.97687
-7.58519	-475.061	0.17251	0.00157	0.00234	6.95901
-7.48561	-475.06097	0.18808	0.00162	0.0024	6.94161
-7.38609	-475.06095	0.20402	0.00166	0.00243	6.92518
-7.28625	-475.06092	0.22005	0.00168	0.00244	6.90844
-7.18671	-475.0609	0.23613	0.00169	0.00245	6.89235
-7.08717	-475.06087	0.25119	0.0017	0.00245	6.87539
-6.98763	-475.06085	0.26546	0.0017	0.00246	6.85836
-6.88804	-475.06083	0.27934	0.00171	0.00246	6.84159
-6.78852	-475.06081	0.29343	0.00171	0.00247	6.82486
-6.68867	-475.06078	0.30782	0.0017	0.00258	6.8072
-6.58913	-475.06076	0.32234	0.00168	0.00258	6.78957
-6.48953	-475.06074	0.33587	0.00165	0.00259	6.77161
-6.38993	-475.06072	0.34867	0.00163	0.00256	6.75414
-6.29033	-475.0607	0.36136	0.00161	0.00252	6.73696
-6.19076	-475.06068	0.37443	0.00158	0.00261	6.71939
-6.0909	-475.06066	0.38776	0.00153	0.00261	6.70088
-5.99128	-475.06063	0.4014	0.00149	0.00262	6.68257
-5.89164	-475.06061	0.41494	0.00144	0.00262	6.66343
-5.79204	-475.06059	0.42844	0.00138	0.00262	6.64377
-5.69242	-475.06057	0.44178	0.00133	0.00263	6.62372
-5.59283	-475.06055	0.45484	0.00128	0.00263	6.60356
-5.49296	-475.06053	0.46758	0.00121	0.00263	6.58295
-5.39331	-475.06051	0.48021	0.00115	0.00263	6.56311
-5.29364	-475.06049	0.49251	0.00108	0.00264	6.54379
-5.19394	-475.06047	0.50483	0.00102	0.00264	6.52489
-5.0942	-475.06045	0.51735	9.57E-4	0.00264	6.50666
-4.99445	-475.06043	0.5302	9.13E-4	0.00264	6.48942
-4.89454	-475.06041	0.5434	8.85E-4	0.00265	6.47243
-4.79475	-475.06039	0.55695	8.8E-4	0.00265	6.45628
-4.69495	-475.06036	0.57054	9.02E-4	0.00265	6.44108
-4.59514	-475.06034	0.58403	9.53E-4	0.00266	6.42647
-4.49531	-475.06032	0.59733	0.00103	0.00266	6.41238
-4.39548	-475.0603	0.61046	0.00113	0.00266	6.39912
-4.29555	-475.06028	0.62353	0.00125	0.00266	6.38681
-4.19569	-475.06026	0.63669	0.0014	0.00267	6.37613
-4.0958	-475.06024	0.65002	0.00157	0.00267	6.36716

-3.99594	-475.06022	0.66368	0.00177	0.00268	6.36021
-3.89608	-475.06019	0.67779	0.002	0.00269	6.35501
-3.79627	-475.06017	0.69242	0.00226	0.0027	6.35198
-3.69638	-475.06015	0.7076	0.00256	0.00272	6.35064
-3.59663	-475.06012	0.72366	0.00291	0.00273	6.35119
-3.49694	-475.06009	0.74078	0.00328	0.00277	6.35349
-3.39728	-475.06006	0.75974	0.00369	0.00282	6.35689
-3.2976	-475.06003	0.78193	0.00413	0.0029	6.36176
-3.19786	-475.05998	0.8093	0.0046	0.00301	6.36756
-3.09801	-475.05993	0.84392	0.00511	0.00314	6.37348
-2.99813	-475.05986	0.88795	0.00566	0.0033	6.37974
-2.89821	-475.05977	0.94333	0.00624	0.00347	6.38557
-2.79826	-475.05966	1.01197	0.00683	0.00367	6.39133
-2.6983	-475.05953	1.09609	0.00745	0.00388	6.39725
-2.59833	-475.05936	1.19868	0.00808	0.00412	6.40357
-2.49836	-475.05917	1.3227	0.00873	0.00437	6.41068
-2.39838	-475.05893	1.47081	0.0094	0.00465	6.41938
-2.29841	-475.05865	1.64551	0.01009	0.00495	6.43029
-2.19843	-475.05833	1.84924	0.01081	0.00528	6.44422
-2.09846	-475.05795	2.08448	0.01155	0.00564	6.46205
-1.99849	-475.05752	2.3549	0.01231	0.00588	6.48464
-1.89853	-475.05703	2.66577	0.01313	0.0063	6.51273
-1.79857	-475.05645	3.02394	0.01402	0.00683	6.54728
-1.69861	-475.0558	3.43698	0.01498	0.00726	6.588
-1.59865	-475.05504	3.91267	0.01602	0.00777	6.63397
-1.49869	-475.05417	4.45881	0.01715	0.00821	6.68585
-1.39873	-475.05317	5.08377	0.01835	0.00879	6.7444
-1.29877	-475.05204	5.79681	0.01963	0.00981	6.8093
-1.19881	-475.05074	6.60916	0.02104	0.01187	6.88204
-1.09887	-475.04926	7.53609	0.02259	0.01294	6.96159
-0.99895	-475.04757	8.6008	0.02443	0.01425	7.05013
-0.89905	-475.04559	9.83922	0.02679	0.01586	7.14465
-0.79919	-475.04325	11.31014	0.0298	0.01796	7.24771
-0.69938	-475.04037	13.11409	0.03393	0.02073	7.35238
-0.59979	-475.03662	15.47006	0.03977	0.02439	7.41691
-0.49988	-475.03132	18.79923	0.04833	0.02916	7.31593
-0.3999	-475.02451	23.06988	0.06226	0.03583	7.03361
-0.29993	-475.01734	27.56645	0.08575	0.04514	6.5599
-0.19996	-475.01114	31.45889	0.12299	0.05749	5.93673
-0.1	-475.00696	34.08235	0.18053	0.07304	5.30584
4E-5	-475.00547	35.01437	0.26884	0.09184	4.89498
0.1	-475.00701	34.05304	0.37617	0.11345	4.91209
0.19996	-475.01152	31.21862	0.46591	0.13788	5.36885
0.29993	-475.01869	26.72324	0.53012	0.16483	6.04486
0.39991	-475.02781	20.99928	0.57963	0.195	6.67792
0.49988	-475.03803	14.58389	0.80639	0.42619	7.10943
0.59985	-475.04856	7.97759	0.82677	0.45395	7.30066
0.69979	-475.05878	1.56717	0.83161	0.46791	7.28241
0.79973	-475.06835	-4.43904	0.83333	0.47977	7.11542
0.89965	-475.07719	-9.98555	0.83374	0.49056	6.8623
0.99956	-475.08532	-15.08823	0.8331	0.50015	6.56558
1.09944	-475.09287	-19.82998	0.83146	0.50834	6.25321
1.19932	-475.10011	-24.37162	0.82901	0.51517	5.96418
1.2992	-475.10717	-28.79784	0.82611	0.52098	5.74053
1.39913	-475.11369	-32.89004	0.8232	0.52577	5.60417
1.49905	-475.11896	-36.19785	0.82056	0.52965	5.5556
1.59881	-475.1226	-38.4819	0.81859	0.53227	5.57925
1.69857	-475.12529	-40.17058	0.81777	0.53326	5.63614
1.7984	-475.12744	-41.52024	0.81713	0.53413	5.69269
1.89827	-475.12921	-42.62904	0.81663	0.53478	5.74036
1.99817	-475.13069	-43.56019	0.81623	0.53532	5.7781
2.09808	-475.13196	-44.35542	0.81589	0.53576	5.80915
2.19801	-475.13305	-45.04206	0.81563	0.53609	5.834
2.29794	-475.13401	-45.64008	0.81544	0.53633	5.85314
2.39788	-475.13484	-46.166	0.8153	0.53647	5.86747
2.49782	-475.13559	-46.63346	0.81523	0.53652	5.87696
2.59777	-475.13626	-47.05292	0.8152	0.53649	5.88391
2.69773	-475.13686	-47.43216	0.81519	0.53639	5.88821
2.7977	-475.13741	-47.77651	0.81521	0.53626	5.89038
2.89768	-475.13791	-48.08904	0.81525	0.5361	5.89105
2.99765	-475.13836	-48.37178	0.81529	0.53594	5.89094

3.09763	-475.13877	-48.62727	0.81532	0.53577	5.89058
3.19761	-475.13913	-48.85802	0.81535	0.53561	5.89083
3.29759	-475.13947	-49.06632	0.81537	0.53547	5.8916
3.39757	-475.13977	-49.25509	0.81538	0.53533	5.89332
3.49755	-475.14004	-49.4266	0.81539	0.5352	5.89684
3.59753	-475.14029	-49.58138	0.81539	0.53508	5.90125
3.69751	-475.14051	-49.71892	0.81539	0.53497	5.90553
3.79748	-475.1407	-49.83933	0.81538	0.53488	5.90959
3.89745	-475.14086	-49.94341	0.81537	0.53481	5.91358
3.99741	-475.14101	-50.03344	0.81536	0.53474	5.91812
4.09737	-475.14113	-50.11225	0.81534	0.53465	5.92406
4.19732	-475.14124	-50.18156	0.81533	0.53452	5.93112
4.29727	-475.14134	-50.24159	0.81533	0.53443	5.93824
4.39721	-475.14142	-50.29222	0.81533	0.53434	5.94551
4.49711	-475.14149	-50.33425	0.81532	0.53428	5.9533
4.59693	-475.14154	-50.36933	0.81532	0.53424	5.96238
4.69669	-475.14159	-50.39954	0.81531	0.53424	5.97265
4.79643	-475.14163	-50.42665	0.81529	0.53424	5.98352
4.89614	-475.14167	-50.45196	0.81528	0.53425	5.99347
4.99605	-475.14171	-50.47554	0.81527	0.53426	6.00283
5.0958	-475.14175	-50.49767	0.81526	0.53427	6.0095
5.19552	-475.14178	-50.51844	0.81526	0.53428	6.01362
5.29523	-475.14181	-50.538	0.81525	0.5343	6.01474
5.39492	-475.14184	-50.5566	0.81525	0.53432	6.01305
5.4946	-475.14187	-50.57455	0.81524	0.53435	6.00795
5.5945	-475.1419	-50.59169	0.81524	0.53442	6.00142
5.69419	-475.14192	-50.60826	0.81524	0.53444	5.99276
5.79389	-475.14195	-50.62419	0.81525	0.53447	5.98234
5.89357	-475.14197	-50.6394	0.81525	0.53449	5.97033
5.99325	-475.142	-50.65374	0.81526	0.53451	5.95648
6.0929	-475.14202	-50.66717	0.81527	0.53453	5.93996
6.19277	-475.14204	-50.67927	0.81529	0.53455	5.92212
6.29238	-475.14205	-50.69021	0.81532	0.53457	5.90146
6.39198	-475.14207	-50.69995	0.81534	0.53458	5.87833
6.49151	-475.14208	-50.70861	0.81537	0.5346	5.85334
6.59095	-475.1421	-50.71644	0.81539	0.53462	5.82684
6.69032	-475.14211	-50.72394	0.81542	0.53464	5.79862
6.79012	-475.14212	-50.7309	0.81545	0.53465	5.76968
6.88951	-475.14213	-50.73779	0.81547	0.53467	5.73948
6.98887	-475.14214	-50.74466	0.8155	0.53468	5.70833
7.08817	-475.14215	-50.75162	0.81553	0.5347	5.67594
7.18742	-475.14216	-50.75874	0.81555	0.53472	5.64295
7.28658	-475.14217	-50.76644	0.81559	0.53473	5.60639
7.38636	-475.14219	-50.77415	0.81563	0.53475	5.56894
7.48572	-475.1422	-50.78237	0.81567	0.53477	5.53123
7.58514	-475.14221	-50.791	0.81571	0.53478	5.49309
7.68449	-475.14223	-50.80006	0.81575	0.5348	5.45612
7.78387	-475.14224	-50.80902	0.8158	0.5348	5.41769
7.88268	-475.14226	-50.81916	0.81583	0.53478	5.37938
7.9825	-475.14227	-50.82874	0.81588	0.53479	5.34139
8.08194	-475.14229	-50.83857	0.81592	0.5348	5.30406
8.18137	-475.14231	-50.84854	0.81597	0.53481	5.2664
8.28071	-475.14232	-50.85875	0.81601	0.53482	5.23027
8.38015	-475.14234	-50.86875	0.81606	0.53483	5.19124
8.47893	-475.14236	-50.88049	0.81611	0.53484	5.15098
8.57874	-475.14238	-50.8926	0.81617	0.53485	5.1098
8.67817	-475.1424	-50.90623	0.81623	0.53485	5.0678
8.77768	-475.14242	-50.92071	0.81629	0.53485	5.02623
8.87715	-475.14244	-50.93537	0.81634	0.53485	4.98675
8.97679	-475.14247	-50.94956	0.8164	0.53485	4.94731
9.07611	-475.14249	-50.96399	0.81646	0.53483	4.90816
9.17591	-475.14251	-50.97808	0.81653	0.53482	4.8708
9.2754	-475.14254	-50.99256	0.8166	0.53481	4.83533
9.37501	-475.14256	-51.00724	0.81667	0.5348	4.80045
9.47459	-475.14258	-51.02206	0.81675	0.53478	4.76777
9.57427	-475.14261	-51.03656	0.81682	0.53477	4.73393
9.67357	-475.14263	-51.05169	0.8169	0.53474	4.70118
9.77344	-475.14265	-51.0667	0.81697	0.53471	4.66983
9.87306	-475.14268	-51.08236	0.81705	0.53469	4.63939
9.97273	-475.1427	-51.09866	0.81712	0.53466	4.60996
10.07242	-475.14273	-51.11568	0.8172	0.53464	4.58226

10.17216	-475.14276	-51.13325	0.81728	0.53462	4.55592
10.27188	-475.14279	-51.15153	0.81736	0.5346	4.52981
10.37178	-475.14282	-51.171	0.81745	0.53458	4.50545
10.4715	-475.14285	-51.19307	0.81754	0.53456	4.48178
10.57128	-475.14289	-51.21771	0.81764	0.53453	4.45818
10.67105	-475.14293	-51.243	0.81774	0.53451	4.43472
10.77085	-475.14297	-51.26765	0.81784	0.5345	4.41212
10.87065	-475.14301	-51.29246	0.81794	0.53448	4.38987
10.97056	-475.14305	-51.31783	0.81805	0.53446	4.36883
11.07033	-475.1431	-51.34432	0.81816	0.53443	4.34891
11.17013	-475.14314	-51.37161	0.81827	0.5344	4.33045
11.26993	-475.14318	-51.39943	0.81837	0.53437	4.31298
11.36974	-475.14323	-51.42721	0.81848	0.53434	4.29721
11.46951	-475.14327	-51.45541	0.8186	0.53432	4.28055
11.56943	-475.14332	-51.48365	0.81873	0.53429	4.26566
11.6692	-475.14336	-51.51221	0.81886	0.53426	4.25156
11.76898	-475.14341	-51.54133	0.81899	0.53424	4.23865
11.86872	-475.14346	-51.56986	0.81913	0.53421	4.22576
11.96846	-475.1435	-51.59682	0.81925	0.53417	4.21259
12.06816	-475.14354	-51.6222	0.81937	0.53413	4.19977
12.16804	-475.14358	-51.64568	0.81949	0.53409	4.18808
12.2677	-475.14361	-51.66744	0.8196	0.53406	4.17611
12.36734	-475.14364	-51.68808	0.81971	0.53403	4.16383
12.46693	-475.14368	-51.70791	0.81981	0.53401	4.15123
12.56651	-475.14371	-51.72684	0.8199	0.53398	4.13622
12.66595	-475.14374	-51.74542	0.81998	0.53395	4.11697
12.76581	-475.14376	-51.76339	0.82004	0.53392	4.09644
12.86539	-475.14379	-51.78125	0.8201	0.53389	4.07428
12.96497	-475.14382	-51.79889	0.82016	0.53386	4.05161
13.06458	-475.14385	-51.8162	0.82021	0.53382	4.02871
13.1642	-475.14387	-51.8332	0.82026	0.53378	4.00577
13.26382	-475.1439	-51.85028	0.8203	0.53375	3.98229
13.36369	-475.14393	-51.86747	0.82034	0.53371	3.95942
13.4633	-475.14396	-51.88538	0.82037	0.53367	3.93635
13.56295	-475.14399	-51.90405	0.8204	0.53363	3.91363
13.66261	-475.14402	-51.92346	0.82042	0.53359	3.89193
13.76229	-475.14405	-51.94351	0.82049	0.53353	3.87132
13.86197	-475.14408	-51.96431	0.82048	0.53351	3.85215
13.96184	-475.14412	-51.98558	0.8205	0.53346	3.83535
14.06148	-475.14415	-52.0076	0.82052	0.53343	3.82036
14.16117	-475.14419	-52.03003	0.82055	0.53339	3.80653
14.26087	-475.14422	-52.05268	0.82056	0.53335	3.79372
14.36063	-475.14426	-52.07532	0.82058	0.5333	3.78219
14.46033	-475.1443	-52.09814	0.82059	0.53325	3.77237
14.56023	-475.14433	-52.1209	0.8206	0.5332	3.76532
14.65994	-475.14437	-52.1441	0.82061	0.53315	3.76037
14.75969	-475.14441	-52.16794	0.82062	0.53311	3.75736
14.85941	-475.14445	-52.19256	0.82062	0.53306	3.75571
14.95916	-475.14449	-52.21793	0.82063	0.53301	3.75529
15.05887	-475.14453	-52.24409	0.82063	0.53296	3.75543
15.15877	-475.14457	-52.27034	0.82063	0.53288	3.75747
15.25848	-475.14461	-52.29688	0.82062	0.53283	3.76047
15.35823	-475.14466	-52.32376	0.82061	0.53277	3.76539
15.45798	-475.1447	-52.35121	0.8206	0.5327	3.77191
15.55777	-475.14475	-52.37925	0.8206	0.53263	3.77994
15.65754	-475.14479	-52.40792	0.82058	0.53255	3.78843
15.75744	-475.14484	-52.43687	0.82056	0.53247	3.79878
15.85716	-475.14488	-52.46652	0.82054	0.53238	3.80986
15.95694	-475.14493	-52.49723	0.82052	0.53229	3.82277
16.05671	-475.14498	-52.52944	0.8205	0.53221	3.83757
16.15653	-475.14504	-52.56297	0.82047	0.53212	3.85418
16.25635	-475.14509	-52.59748	0.82044	0.53202	3.8719
16.35627	-475.14515	-52.63234	0.82041	0.53193	3.89167
16.45608	-475.1452	-52.66774	0.82037	0.53183	3.91229
16.55591	-475.14526	-52.70366	0.82033	0.53173	3.93379
16.65574	-475.14532	-52.74045	0.82029	0.53162	3.95621
16.75557	-475.14538	-52.77825	0.82024	0.53151	3.97973
16.85539	-475.14544	-52.81746	0.82019	0.53139	4.00474
16.95533	-475.14551	-52.85837	0.82014	0.53127	4.03233
17.05516	-475.14558	-52.90163	0.82008	0.53114	4.06085
17.15501	-475.14565	-52.94659	0.82002	0.531	4.0898

17.25486	-475.14572	-52.99207	0.81996	0.53086	4.11823
17.35472	-475.14579	-53.03759	0.8199	0.53071	4.14713
17.45457	-475.14587	-53.08369	0.81982	0.53054	4.17741
17.55452	-475.14594	-53.13042	0.81975	0.53037	4.20954
17.65437	-475.14602	-53.17668	0.81968	0.53019	4.24113
17.75422	-475.14609	-53.22073	0.81961	0.53001	4.27094
17.85407	-475.14615	-53.2627	0.81953	0.52982	4.30176
17.95393	-475.14622	-53.30234	0.81946	0.52963	4.33261
18.05376	-475.14628	-53.34113	0.81937	0.52943	4.36579
18.15371	-475.14634	-53.37855	0.81928	0.52923	4.3997
18.25354	-475.14639	-53.41348	0.8192	0.52903	4.43061
18.35337	-475.14644	-53.4452	0.81912	0.52884	4.46008
18.45314	-475.14649	-53.47297	0.81903	0.52867	4.48608
18.55283	-475.14653	-53.4971	0.81895	0.52851	4.50926
18.65241	-475.14656	-53.51825	0.81888	0.52837	4.52899
18.75222	-475.14659	-53.53609	0.81882	0.52826	4.54348
18.85155	-475.14661	-53.55143	0.81876	0.52817	4.55152
18.95067	-475.14663	-53.56473	0.81872	0.52811	4.55558
19.04962	-475.14665	-53.57642	0.81869	0.52806	4.55728
19.14854	-475.14667	-53.58658	0.81865	0.52801	4.5572
19.24741	-475.14668	-53.59582	0.81864	0.528	4.55651
19.34679	-475.1467	-53.60287	0.81861	0.52796	4.55928
19.44514	-475.1467	-53.60903	0.81858	0.52791	4.56434
19.54339	-475.14671	-53.61397	0.81854	0.52786	4.57235
19.64138	-475.14672	-53.61803	0.8185	0.5278	4.58412
19.73904	-475.14672	-53.62121	0.81847	0.52775	4.59936
19.83621	-475.14673	-53.62371	0.81846	0.52774	4.61437

[a]Referenced to -475.06127353 hartree.

Table S6: Data shown in Figures 3 and 4 for prop-1-ene.

**Reaction Coordinate [bohr amu$^{1/2}$]**	**E [hartree]**	**ΔE [kcal mol^{-1}]^{a}**	**IBO Changes for π-Bond Olefin**	**IBO Changes for Lone Pair on Oxygen**	**Dipole Moment [debye]**
-5.98506	-422.12048	0.36216	0	0	2.33573
-5.88684	-422.12048	0.36276	1.2E-5	6E-6	2.33191
-5.78897	-422.12048	0.3636	4.9E-5	4E-6	2.32672
-5.69041	-422.12048	0.3648	1.17E-4	1.2E-5	2.32082
-5.59078	-422.12047	0.36662	2.03E-4	1.5E-5	2.31512
-5.49144	-422.12047	0.36939	2.77E-4	1.9E-5	2.31005
-5.39215	-422.12046	0.37292	3.63E-4	2.6E-5	2.30471
-5.2928	-422.12046	0.37719	4.34E-4	3.2E-5	2.30021
-5.19348	-422.12045	0.38209	5.05E-4	3.6E-5	2.2963
-5.09371	-422.12044	0.38728	5.7E-4	3.9E-5	2.293
-4.99444	-422.12043	0.39277	6.36E-4	4.4E-5	2.29037
-4.89514	-422.12042	0.3982	7.45E-4	5.1E-5	2.28758
-4.79572	-422.12041	0.4041	8.74E-4	5.9E-5	2.28559
-4.69615	-422.1204	0.41098	0.00102	6.7E-5	2.28461
-4.59636	-422.12039	0.41931	0.0012	7.4E-5	2.28466
-4.49663	-422.12037	0.42966	0.00139	8.1E-5	2.28567
-4.39679	-422.12035	0.44266	0.0016	8.4E-5	2.2877
-4.29688	-422.12032	0.45899	0.00182	8.6E-5	2.29027
-4.19695	-422.12029	0.47892	0.00205	8.6E-5	2.2937
-4.09699	-422.12026	0.50239	0.00229	8.5E-5	2.29775
-3.99704	-422.12021	0.52932	0.00252	8.3E-5	2.30236
-3.89708	-422.12016	0.55956	0.00275	7.9E-5	2.30741
-3.79713	-422.12011	0.59287	0.00299	7.3E-5	2.3128
-3.69717	-422.12005	0.62898	0.00321	6.7E-5	2.3182
-3.5972	-422.11999	0.66758	0.00344	5.8E-5	2.32339
-3.49725	-422.11993	0.70858	0.00365	5.2E-5	2.32848
-3.39731	-422.11986	0.75202	0.00385	4.9E-5	2.3333
-3.29738	-422.11978	0.79821	0.00403	5.9E-5	2.33771
-3.19746	-422.11971	0.84766	0.00419	8.7E-5	2.34193
-3.09754	-422.11962	0.90107	0.00434	1.33E-4	2.34592
-2.99764	-422.11953	0.95936	0.00446	1.95E-4	2.34977
-2.89774	-422.11943	1.0236	0.00456	2.68E-4	2.35333
-2.79784	-422.11931	1.09547	0.00465	3.68E-4	2.35702
-2.69794	-422.11918	1.17701	0.00472	4.86E-4	2.36093
-2.59801	-422.11903	1.27047	0.00479	6.18E-4	2.36542
-2.49807	-422.11886	1.37843	0.00486	7.68E-4	2.37053
-2.39812	-422.11866	1.50384	0.00495	9.36E-4	2.37703

-2.29817	-422.11843	1.64971	0.00506	0.00112	2.38544
-2.1982	-422.11816	1.81851	0.0052	0.00132	2.396
-2.09823	-422.11785	2.01223	0.00537	0.00153	2.40917
-1.99827	-422.1175	2.23303	0.00558	0.00177	2.42538
-1.8983	-422.1171	2.48346	0.00583	0.00202	2.44535
-1.79834	-422.11665	2.76729	0.00612	0.00229	2.46974
-1.69838	-422.11613	3.08956	0.00646	0.00259	2.49913
-1.59843	-422.11555	3.45629	0.00683	0.00291	2.53369
-1.49848	-422.11488	3.87466	0.00724	0.00326	2.57257
-1.39853	-422.11412	4.35318	0.00768	0.00363	2.61415
-1.29857	-422.11325	4.90103	0.00815	0.00404	2.65861
-1.19862	-422.11225	5.5282	0.00866	0.00448	2.70591
-1.09866	-422.1111	6.24653	0.00921	0.00497	2.75477
-0.99874	-422.10979	7.07099	0.00989	0.00549	2.80036
-0.89883	-422.10828	8.02003	0.01058	0.00619	2.84994
-0.79896	-422.10651	9.13064	0.01177	0.00695	2.87606
-0.69915	-422.10438	10.46478	0.01318	0.00781	2.89974
-0.5995	-422.10169	12.15528	0.0165	0.009	2.84735
-0.49986	-422.09781	14.58622	0.02434	0.01072	2.61556
-0.3999	-422.09234	18.01996	0.03961	0.01296	2.10608
-0.29993	-422.08604	21.9718	0.0644	0.01654	1.31514
-0.19996	-422.08025	25.60403	0.10206	0.0221	0.59041
-0.1	-422.07623	28.12761	0.15739	0.0303	1.66644
0	-422.07478	29.03669	0.23736	0.04166	3.33241
0.1	-422.0763	28.08446	0.33682	0.0565	5.10402
0.19996	-422.08081	25.25231	0.42834	0.07506	6.8052
0.29993	-422.08807	20.70224	0.49849	0.0975	8.27118
0.39991	-422.09744	14.81989	0.55196	0.12415	9.37126
0.49988	-422.10812	8.12039	0.59758	0.1581	10.06743
0.59986	-422.11928	1.11212	0.65398	0.21734	10.40763
0.69983	-422.13016	-5.71347	0.73879	0.31355	10.46871
0.79981	-422.14004	-11.91487	0.78567	0.36939	10.32015
0.89975	-422.14838	-17.14432	0.80364	0.39781	10.02135
0.99962	-422.15487	-21.21915	0.81165	0.41777	9.62525
1.09927	-422.15972	-24.26276	0.81561	0.43315	9.1936
1.19893	-422.16353	-26.65294	0.81731	0.44452	8.75635
1.29869	-422.16695	-28.8015	0.81717	0.45184	8.31878
1.39857	-422.17041	-30.97282	0.81566	0.45773	7.83096
1.49851	-422.17415	-33.31626	0.81331	0.46364	7.26942
1.59843	-422.17836	-35.95597	0.81023	0.47001	6.61521
1.69838	-422.18308	-38.91906	0.80693	0.47631	5.93054
1.79834	-422.18801	-42.01499	0.80364	0.48235	5.26194
1.8983	-422.1925	-44.83299	0.80062	0.48782	4.66439
1.9982	-422.19595	-46.99397	0.79816	0.49226	4.20861
2.09793	-422.19849	-48.59271	0.79678	0.4947	4.01333
2.19776	-422.2005	-49.85473	0.79594	0.49607	3.92471
2.29762	-422.20215	-50.88865	0.79535	0.497	3.88073
2.39753	-422.20353	-51.75313	0.79487	0.49775	3.84679
2.49744	-422.20469	-52.48383	0.79452	0.49827	3.83185
2.59735	-422.20569	-53.10624	0.79425	0.49868	3.81793
2.69727	-422.20654	-53.6403	0.79408	0.49894	3.81536
2.79719	-422.20728	-54.10371	0.79398	0.49907	3.81971
2.89712	-422.20793	-54.5112	0.79395	0.49911	3.83106
2.99707	-422.2085	-54.87407	0.79397	0.49906	3.84778
3.09702	-422.20903	-55.20169	0.79402	0.49897	3.86766
3.19699	-422.2095	-55.49984	0.7941	0.49885	3.89064
3.29696	-422.20993	-55.77051	0.79422	0.4986	3.91412
3.39694	-422.21032	-56.01461	0.7943	0.49848	3.93785
3.49692	-422.21067	-56.23425	0.79437	0.49837	3.96123
3.5969	-422.21099	-56.4317	0.79443	0.49829	3.98377
3.69687	-422.21127	-56.60792	0.79444	0.49836	4.00534
3.79685	-422.21152	-56.76379	0.79447	0.49832	4.02444
3.89682	-422.21173	-56.90014	0.79449	0.49831	4.0419
3.99678	-422.21192	-57.01873	0.7945	0.49832	4.05885
4.09673	-422.21209	-57.12228	0.79449	0.49835	4.07606
4.19669	-422.21223	-57.21219	0.79448	0.49838	4.09485
4.29663	-422.21235	-57.28945	0.79446	0.49841	4.1154
4.39655	-422.21246	-57.35551	0.79445	0.49843	4.139
4.49646	-422.21255	-57.41271	0.79444	0.49844	4.16599
4.59635	-422.21263	-57.46257	0.79444	0.49845	4.1957
4.69627	-422.2127	-57.50613	0.79444	0.49844	4.22754

4.79613	-422.21276	-57.54471	0.79443	0.49844	4.25898
4.89597	-422.21281	-57.57929	0.7944	0.49845	4.28981
4.99577	-422.21287	-57.61095	0.79438	0.49846	4.32013
5.09556	-422.21291	-57.64089	0.79434	0.49848	4.34894
5.19548	-422.21296	-57.66963	0.79432	0.49848	4.37873
5.29531	-422.213	-57.69759	0.7943	0.49847	4.4074
5.39513	-422.21305	-57.72481	0.79429	0.49846	4.43566
5.49496	-422.21309	-57.75126	0.79428	0.49844	4.46368
5.59479	-422.21313	-57.77693	0.79427	0.49842	4.49014
5.69473	-422.21317	-57.80154	0.79427	0.49838	4.518
5.79454	-422.21321	-57.82514	0.79427	0.49835	4.54477
5.89435	-422.21324	-57.84764	0.79427	0.49832	4.57075
5.99415	-422.21328	-57.86904	0.79427	0.4983	4.59613
6.09396	-422.21331	-57.88943	0.79426	0.49829	4.61981
6.19388	-422.21334	-57.90875	0.79426	0.49826	4.64461
6.29366	-422.21337	-57.92729	0.79425	0.49824	4.66834
6.39344	-422.2134	-57.94511	0.79424	0.49823	4.6913
6.49321	-422.21343	-57.96228	0.79423	0.49823	4.71346
6.593	-422.21345	-57.97888	0.79422	0.49824	4.73381
6.69292	-422.21348	-57.99473	0.79421	0.49823	4.75492
6.79269	-422.2135	-58.00995	0.79421	0.49822	4.77512
6.89246	-422.21352	-58.02446	0.79424	0.49809	4.79464
6.9922	-422.21355	-58.03822	0.79424	0.49807	4.8135
7.09193	-422.21357	-58.0512	0.79424	0.49806	4.83043
7.19185	-422.21359	-58.06313	0.7942	0.49816	4.84815
7.29157	-422.2136	-58.07405	0.7942	0.49813	4.86517
7.39128	-422.21362	-58.08381	0.7942	0.49809	4.88127
7.49096	-422.21363	-58.09229	0.79425	0.49793	4.89657
7.5906	-422.21364	-58.09942	0.79424	0.4979	4.9102
7.69045	-422.21365	-58.10501	0.7942	0.498	4.92428
7.78998	-422.21366	-58.10932	0.79424	0.49784	4.93685
7.88943	-422.21366	-58.11262	0.79424	0.49781	4.94704
7.98881	-422.21367	-58.11517	0.79424	0.49779	4.95468
8.08802	-422.21367	-58.11717	0.79419	0.49791	4.95923

[a]Referenced to -422.121056397 hartree.

Table S7: Data shown in Figures 3 and 4 for hydroxyethene.

Reaction Coordinate [bohr amu$^{1/2}$]	E [hartree]	ΔE [kcal mol^{-1}][a]	IBO Changes for π-Bond Olefin	IBO Changes for Lone Pair on Oxygen	Dipole Moment [debye]
-8.66429	-458.04696	0.00464	0	0	1.9069
-8.56616	-458.04696	0.00556	8E-5	3E-6	1.89505
-8.46786	-458.04696	0.00652	1.77E-4	8E-6	1.88202
-8.36967	-458.04696	0.00793	2.32E-4	1.1E-5	1.87126
-8.2708	-458.04695	0.01002	3.27E-4	1.5E-5	1.86257
-8.17185	-458.04695	0.01299	4E-4	1.7E-5	1.85777
-8.07226	-458.04694	0.0165	4.92E-4	1.8E-5	1.85632
-7.97324	-458.04694	0.02046	4.94E-4	1.7E-5	1.85752
-7.87422	-458.04693	0.02387	5.52E-4	2E-5	1.85634
-7.77514	-458.04693	0.02644	6.24E-4	2.7E-5	1.85425
-7.67555	-458.04692	0.02813	7.4E-4	2.4E-5	1.8513
-7.57685	-458.04692	0.02963	8.14E-4	1.51E-4	1.8492
-7.47766	-458.04692	0.03121	9.09E-4	1.5E-4	1.84374
-7.3786	-458.04692	0.03337	9.82E-4	1.49E-4	1.83964
-7.27898	-458.04691	0.03599	0.00105	1.48E-4	1.83852
-7.17991	-458.04691	0.03927	0.00102	1.48E-4	1.84024
-7.08075	-458.0469	0.04276	9.97E-4	1.47E-4	1.84632
-6.98168	-458.0469	0.04624	9.59E-4	1.47E-4	1.85385
-6.88215	-458.04689	0.0491	9.64E-4	1.46E-4	1.8648
-6.78354	-458.04689	0.05159	9.24E-4	1.46E-4	1.8791
-6.68456	-458.04688	0.05354	8.47E-4	1.45E-4	1.89461
-6.58537	-458.04688	0.05589	7.28E-4	1.45E-4	1.91206
-6.48566	-458.04688	0.05876	6.19E-4	1.44E-4	1.92873
-6.38633	-458.04687	0.06245	5.2E-4	1.44E-4	1.94571
-6.28691	-458.04686	0.0666	4.27E-4	1.43E-4	1.96328
-6.18746	-458.04686	0.07126	3.75E-4	1.42E-4	1.98118
-6.08765	-458.04685	0.07604	3.81E-4	1.42E-4	1.99964
-5.98829	-458.04684	0.08098	4.09E-4	1.41E-4	2.0195
-5.88884	-458.04683	0.08585	4.6E-4	1.4E-4	2.04084
-5.78951	-458.04682	0.09105	5.37E-4	1.39E-4	2.06094

-5.68979	-458.04681	0.09693	6.3E-4	1.39E-4	2.0814
-5.59047	-458.0468	0.10415	7.26E-4	1.38E-4	2.10282
-5.49108	-458.04679	0.11228	8.45E-4	1.38E-4	2.12309
-5.39171	-458.04678	0.12109	9.55E-4	1.39E-4	2.14312
-5.29192	-458.04676	0.13034	0.00103	1.41E-4	2.16519
-5.19268	-458.04675	0.14	0.00109	1.44E-4	2.19096
-5.09343	-458.04673	0.14892	0.00115	1.5E-4	2.21353
-4.99413	-458.04672	0.15723	0.00123	1.58E-4	2.23466
-4.8944	-458.04671	0.16527	0.00134	1.67E-4	2.25588
-4.79505	-458.04669	0.17407	0.00148	1.77E-4	2.27861
-4.69556	-458.04668	0.18398	0.00164	1.89E-4	2.30063
-4.596	-458.04666	0.19567	0.00181	2.01E-4	2.32259
-4.49612	-458.04664	0.20922	0.002	2.15E-4	2.346
-4.3964	-458.04661	0.22481	0.0022	2.29E-4	2.36892
-4.29658	-458.04658	0.24218	0.00241	2.42E-4	2.39073
-4.19671	-458.04655	0.26145	0.00265	2.53E-4	2.4112
-4.0968	-458.04652	0.28272	0.0029	2.62E-4	2.43031
-3.99689	-458.04648	0.30624	0.00316	2.67E-4	2.44735
-3.89697	-458.04644	0.33228	0.00343	2.69E-4	2.46275
-3.79704	-458.04639	0.3611	0.0037	2.67E-4	2.4761
-3.69708	-458.04634	0.39292	0.004	2.63E-4	2.48736
-3.59712	-458.04629	0.42772	0.0043	2.58E-4	2.49728
-3.49717	-458.04623	0.46513	0.00461	2.52E-4	2.50647
-3.39722	-458.04616	0.50476	0.00495	2.45E-4	2.51565
-3.29725	-458.0461	0.54643	0.00529	2.38E-4	2.52443
-3.1973	-458.04603	0.59024	0.00564	2.32E-4	2.53322
-3.09737	-458.04595	0.63639	0.00599	2.26E-4	2.54211
-2.99744	-458.04588	0.68534	0.00633	2.22E-4	2.55142
-2.89751	-458.04579	0.73777	0.00667	2.19E-4	2.56099
-2.7976	-458.0457	0.79478	0.00699	2.17E-4	2.57101
-2.6977	-458.0456	0.85764	0.00729	2.16E-4	2.58124
-2.59779	-458.04549	0.92802	0.00758	2.17E-4	2.59218
-2.49787	-458.04536	1.00785	0.00785	2.2E-4	2.60444
-2.39795	-458.04522	1.09948	0.00811	2.24E-4	2.61805
-2.29802	-458.04505	1.20514	0.00838	2.5E-4	2.63337
-2.19808	-458.04485	1.32701	0.00867	2.19E-4	2.65016
-2.09812	-458.04463	1.46708	0.009	2.18E-4	2.66888
-1.99817	-458.04438	1.6275	0.00937	2.23E-4	2.6894
-1.89821	-458.04408	1.81104	0.00976	2.3E-4	2.71294
-1.79825	-458.04375	2.021	0.0102	2.38E-4	2.73926
-1.69829	-458.04337	2.2609	0.01067	2.35E-4	2.76908
-1.59833	-458.04293	2.53488	0.01117	3.18E-4	2.80294
-1.49839	-458.04243	2.84863	0.01168	3.4E-4	2.84117
-1.39844	-458.04185	3.21007	0.01219	3.66E-4	2.88345
-1.2985	-458.04119	3.62796	0.01272	3.96E-4	2.92884
-1.19855	-458.04042	4.11172	0.01329	4.3E-4	2.97559
-1.0986	-458.03952	4.67246	0.0139	4.71E-4	3.02183
-0.99868	-458.03848	5.32481	0.01465	5.21E-4	3.06523
-0.8988	-458.03726	6.09039	0.01567	5.87E-4	3.10146
-0.79901	-458.03579	7.01382	0.0172	3.32E-4	3.11929
-0.69929	-458.03393	8.18053	0.01938	4.34E-4	3.11996
-0.59965	-458.03143	9.74945	0.02229	3.97E-4	3.07011
-0.49983	-458.02771	12.08818	0.02718	0.00124	2.82085
-0.3999	-458.02222	15.53074	0.03755	0.0063	2.34753
-0.29993	-458.01582	19.54499	0.0576	0.01215	1.63482
-0.19996	-458.00992	23.24772	0.09059	0.01564	1.19496
-0.1	-458.00584	25.80871	0.14067	0.02066	2.23634
4E-5	-458.00438	26.72224	0.21568	0.02748	4.03079
0.1	-458.00589	25.77942	0.31658	0.03645	6.03071
0.19996	-458.01032	22.99574	0.41965	0.04792	8.01711
0.29993	-458.01737	18.57088	0.50141	0.06237	9.79551
0.39991	-458.02634	12.94673	0.56317	0.08017	11.20106
0.49988	-458.03622	6.74655	0.61205	0.10128	12.1701
0.59986	-458.04596	0.63121	0.65254	0.12498	12.75765
0.69983	-458.0545	-4.72417	0.68597	0.14977	13.02644
0.79977	-458.06085	-8.71039	0.71215	0.17391	13.05034
0.89903	-458.06491	-11.2609	0.72881	0.19585	12.89014
0.99858	-458.06798	-13.18306	0.73666	0.21463	12.6179
1.09845	-458.07086	-14.98928	0.7421	0.23379	12.27474
1.19837	-458.07372	-16.78858	0.74822	0.25537	11.86811
1.2983	-458.07664	-18.62058	0.75505	0.27931	11.40725

1.39825	-458.07966	-20.51268	0.76244	0.30488	10.89906
1.49821	-458.08281	-22.49161	0.77024	0.33162	10.33873
1.59816	-458.08615	-24.58443	0.77774	0.35834	9.72792
1.69811	-458.08971	-26.82282	0.78404	0.38378	9.04747
1.79806	-458.09358	-29.24716	0.78857	0.40637	8.32543
1.898	-458.09786	-31.93234	0.79092	0.42558	7.55189
1.99794	-458.10272	-34.98274	0.79132	0.44142	6.7426
2.09789	-458.10814	-38.38658	0.79037	0.45443	5.93729
2.19784	-458.1137	-41.87545	0.78882	0.46516	5.19478
2.2978	-458.11867	-44.99236	0.78717	0.47387	4.55201
2.39769	-458.12239	-47.32531	0.78568	0.48085	4.06633
2.49738	-458.12505	-48.99845	0.78485	0.48465	3.84496
2.5972	-458.1271	-50.28385	0.78426	0.48659	3.72658
2.69706	-458.12873	-51.30736	0.78375	0.4879	3.64604
2.79695	-458.13005	-52.13496	0.78336	0.48887	3.57566
2.89685	-458.13112	-52.80662	0.78306	0.48959	3.51879
2.99674	-458.13199	-53.35011	0.78285	0.49015	3.47042
3.0966	-458.13269	-53.79027	0.78273	0.49055	3.43164
3.19645	-458.13326	-54.15106	0.78269	0.49078	3.40311
3.29632	-458.13375	-54.45425	0.78273	0.49086	3.38406
3.39624	-458.13416	-54.71616	0.78282	0.49082	3.37449
3.49618	-458.13453	-54.94668	0.78294	0.49072	3.37148
3.59615	-458.13486	-55.15117	0.78311	0.49053	3.37268
3.69612	-458.13515	-55.33289	0.78323	0.49039	3.37732
3.7961	-458.13541	-55.49448	0.78334	0.49024	3.38543
3.89606	-458.13564	-55.63901	0.78339	0.49016	3.3965
3.99603	-458.13584	-55.76826	0.78347	0.49003	3.41098
4.09599	-458.13602	-55.88198	0.78352	0.48992	3.42728
4.19595	-458.13618	-55.97978	0.78355	0.48982	3.44594
4.2959	-458.13631	-56.06322	0.78355	0.48973	3.46692
4.39584	-458.13643	-56.135	0.78354	0.48967	3.49069
4.49577	-458.13652	-56.19695	0.78353	0.48962	3.51526
4.59569	-458.13661	-56.25045	0.7835	0.48957	3.54057
4.69558	-458.13668	-56.29646	0.78347	0.48954	3.56795
4.79542	-458.13675	-56.33588	0.78342	0.4895	3.60041
4.89519	-458.1368	-56.37039	0.78336	0.48946	3.63798
4.99505	-458.13685	-56.40159	0.7833	0.48943	3.67842
5.09479	-458.1369	-56.4309	0.78325	0.48941	3.71872
5.19454	-458.13694	-56.45906	0.7832	0.4894	3.75785
5.29428	-458.13699	-56.4865	0.78315	0.48939	3.79486
5.39419	-458.13703	-56.51299	0.78311	0.48937	3.83091
5.49394	-458.13707	-56.53872	0.78308	0.48936	3.86479
5.59369	-458.13711	-56.56362	0.78305	0.48936	3.89784
5.69343	-458.13715	-56.58768	0.78303	0.48935	3.92958
5.79335	-458.13718	-56.61036	0.78301	0.48934	3.96205
5.89308	-458.13722	-56.6317	0.78299	0.48932	3.99327
5.9928	-458.13725	-56.65175	0.78295	0.48931	4.02363
6.09251	-458.13728	-56.67086	0.7829	0.48931	4.052
6.1924	-458.13731	-56.68906	0.78285	0.4893	4.0809
6.29209	-458.13734	-56.70684	0.78279	0.48929	4.10995
6.39179	-458.13736	-56.72416	0.78272	0.48929	4.13946
6.49146	-458.13739	-56.74094	0.78263	0.48926	4.17025
6.59135	-458.13742	-56.75655	0.78257	0.48924	4.19949
6.69101	-458.13744	-56.77093	0.78254	0.48923	4.22498
6.79069	-458.13746	-56.78391	0.78251	0.4892	4.25179
6.89034	-458.13748	-56.79558	0.7825	0.48919	4.27344
6.99021	-458.13749	-56.80574	0.78254	0.48906	4.29692
7.08983	-458.13751	-56.81461	0.78254	0.48904	4.31938
7.18941	-458.13752	-56.82221	0.78249	0.48912	4.34161
7.28892	-458.13753	-56.82869	0.78246	0.48911	4.36234
7.38861	-458.13754	-56.83394	0.78245	0.48909	4.38133
7.48793	-458.13755	-56.83823	0.78245	0.48907	4.39533
7.58708	-458.13755	-56.84166	0.78244	0.48906	4.40645
7.68613	-458.13756	-56.84446	0.78243	0.48906	4.41149
7.7853	-458.13756	-56.84669	0.78243	0.48906	4.41341

[a]Referenced to -458.046969109 hartree.
